# GSH-responsive degradable nanodrug for glucose metabolism intervention and induction of ferroptosis to enhance magnetothermal anti-tumor therapy

**DOI:** 10.1186/s12951-024-02425-4

**Published:** 2024-04-03

**Authors:** Zhen Liao, E. Wen, Yi Feng

**Affiliations:** 1 Department of Biomedical Engineering, School of Life Science and Technology, University of Electronic Science and Technology of China, Chengdu, 61173 Sichuan People’s Republic of China; 2 Institute of Burn Research Southwest Hospital, Third Military Medical University (Army Medical University), Chongqing, 400038 People’s Republic of China; 3 Precision Medicine Center, The Second Affiliated Hospital of Chongqing Medical University, Chongqing, People’s Republic of China

**Keywords:** Magnetothermal therapy, Ferroptosis, Metabolic interference, Redox homeostasis, Mesoporous silica nanoparticle

## Abstract

The challenges associated with activating ferroptosis for cancer therapy primarily arise from obstacles related to redox and iron homeostasis, which hinder the susceptibility of tumor cells to ferroptosis. However, the specific mechanisms of ferroptosis resistance, especially those intertwined with abnormal metabolic processes within tumor cells, have been consistently underestimated. In response, we present an innovative glutathione-responsive magnetocaloric therapy nanodrug termed LFMP. LFMP consists of lonidamine (LND) loaded into PEG-modified magnetic nanoparticles with a Fe_3_O_4_ core and coated with disulfide bonds-bridged mesoporous silica shells. This nanodrug is designed to induce an accelerated ferroptosis-activating state in tumor cells by disrupting homeostasis. Under the dual effects of alternating magnetic fields and high concentrations of glutathione in the tumor microenvironment, LFMP undergoes disintegration, releasing drugs. LND intervenes in cell metabolism by inhibiting glycolysis, ultimately enhancing iron death and leading to synthetic glutathione consumption. The disulfide bonds play a pivotal role in disrupting intracellular redox homeostasis by depleting glutathione and inactivating glutathione peroxidase 4 (GPX4), synergizing with LND to enhance the sensitivity of tumor cells to ferroptosis. This process intensifies oxidative stress, further impairing redox homeostasis. Furthermore, LFMP exacerbates mitochondrial dysfunction, triggering ROS formation and lactate buildup in cancer cells, resulting in increased acidity and subsequent tumor cell death. Importantly, LFMP significantly suppresses tumor cell proliferation with minimal side effects both in vitro and in vivo, exhibiting satisfactory *T2*-weighted MR imaging properties. In conclusion, this magnetic hyperthermia-based nanomedicine strategy presents a promising and innovative approach for antitumor therapy.

**Graphical Abstract:**

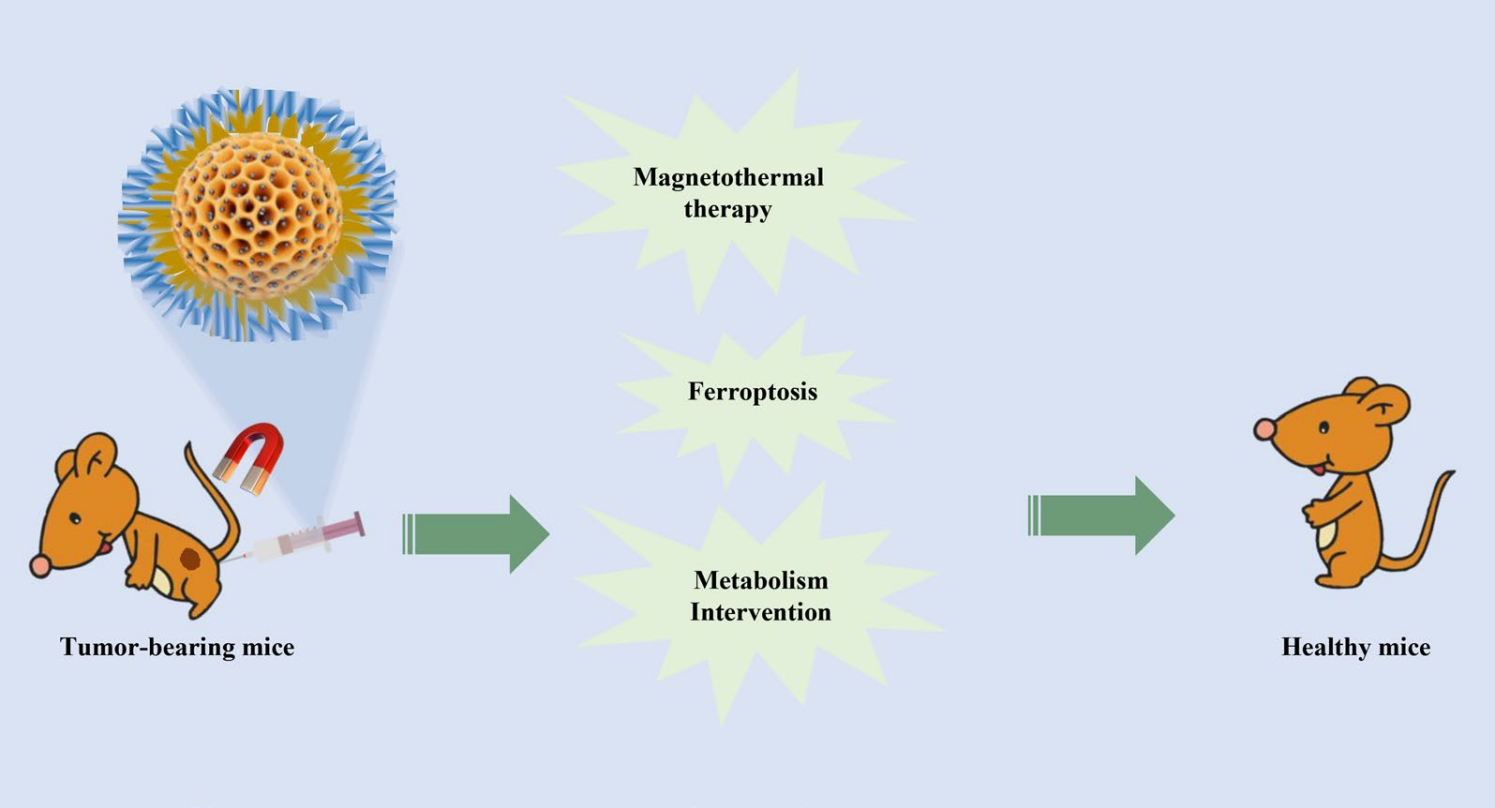

**Supplementary Information:**

The online version contains supplementary material available at 10.1186/s12951-024-02425-4.

## Introduction

In tumor treatment, ferroptosis, a non-apoptotic programmed cell death pathway dependent on iron, holds great promise for synergistic radiotherapy and chemotherapy [1]. This process is characterized by elevated levels of ferric ions, reactive oxygen species (ROS), lipid peroxides, and mitochondrial impairment [2, 3]. Transition metals have been extensively explored to induce ferroptosis, aiming to enhance therapeutic efficacy in tumor treatment [4]. Iron oxide nanoparticles find widespread application in tumor treatment, primarily by instigating ferroptosis [5, 6]. However, the inherent self-regulation of redox and iron homeostasis within tumor cells significantly hampers the efficacy of antitumor interventions [7]. Therefore, it is essential to strategically integrate disruption of redox and iron homeostasis to synergize apoptosis and ferroptosis, thereby reinforcing antitumor efficiency.

The central event in the ferroptosis process is the generation of lipid peroxides, directly propelled by reactive oxygen species (ROS) [8]. To regulate lipid peroxide levels in tumors, redox homeostasis plays a crucial role through the abundance of glutathione (GSH) and the synthesis or expression of glutathione peroxidase 4 (GPX4) [9]. In tumors, the overexpression of GSH serves a dual purpose: scavenging ROS and oxidizing GSH to oxidized glutathione (GSSH) [10–12]. Numerous studies have confirmed that the activation of GPX4 relies on GSH assistance, establishing the GSH/GPX4 system as the central regulatory mechanism in ferroptosis [13]. However, previous research has primarily focused on accelerating ROS generation for tumor treatment, often overlooking the impact of intrinsic ferroptosis resistance mechanisms in tumors [14–16]. Consequently, a more effective antitumor therapy approach involves exploring drugs or mechanisms that deplete GSH, thereby inactivating GPX4 and inducing ferroptosis through the mediation of redox homeostasis in tumor cells.

Reducing proliferation in tumor cells emerges as a pivotal pathway by simultaneously inhibiting the self-maintenance of glucose metabolism and disrupting intracellular redox homeostasis [17, 18]. The bioenergetic and biosynthetic processes in tumor cells necessitate reprogrammed metabolic behaviors, which are susceptible to interference from genetic mutations, contributing to the evasion of metabolic monitoring and uncontrolled proliferation [19–21]. Studies have abundantly demonstrated the profound impact of glucose metabolism on the pathogenesis and progression of tumors [22]. In the spectrum of glucose metabolism processes, tumor cells predominantly utilize aerobic glycolysis to generate ample adenosine triphosphate (ATP) [23, 24]. Glucose undergoes conversion into glucose-6-phosphate (G6P), serving as an energy source in the body and undergoing metabolism through glycolysis and the pentose phosphate pathway (PPP) [25]. Notably, PPP flux plays a crucial role in resisting various treatments in tumor cells, influencing the recycling of oxidized glutathione (GSH) [26]. This suggests that suppressing PPP is an effective approach for tumor cells to enhance ferroptosis through GSH-regulated sensitivity [27]. Therefore, concurrently regulating glycolysis and PPP is advisable for exploring anti-tumor agents via inducing ferroptosis."

Recently, Lonidamine (LND) has been reported as a favorable anti-tumor medicine due to its impact on mitochondria, glycolysis, the inhibition of GSH, and PPP [28–31]. The combination of anticancer drugs with chemotherapy, photodynamic therapy, or sonodynamic therapy demonstrates a synergistic therapeutic effect in tumors [32]. Notably, magnetic hyperthermia has gained widespread attention as a potential therapeutic method for tumors [33]. In this context, we utilized LND to engineer a multifunctional nanomedicine (designated as LND@Fe3O4@MONs-PEG, LFMP), regulating glycolysis and ferroptosis for antitumor efficacy by impairing homeostatic redox and iron balance.

**Scheme 1:**
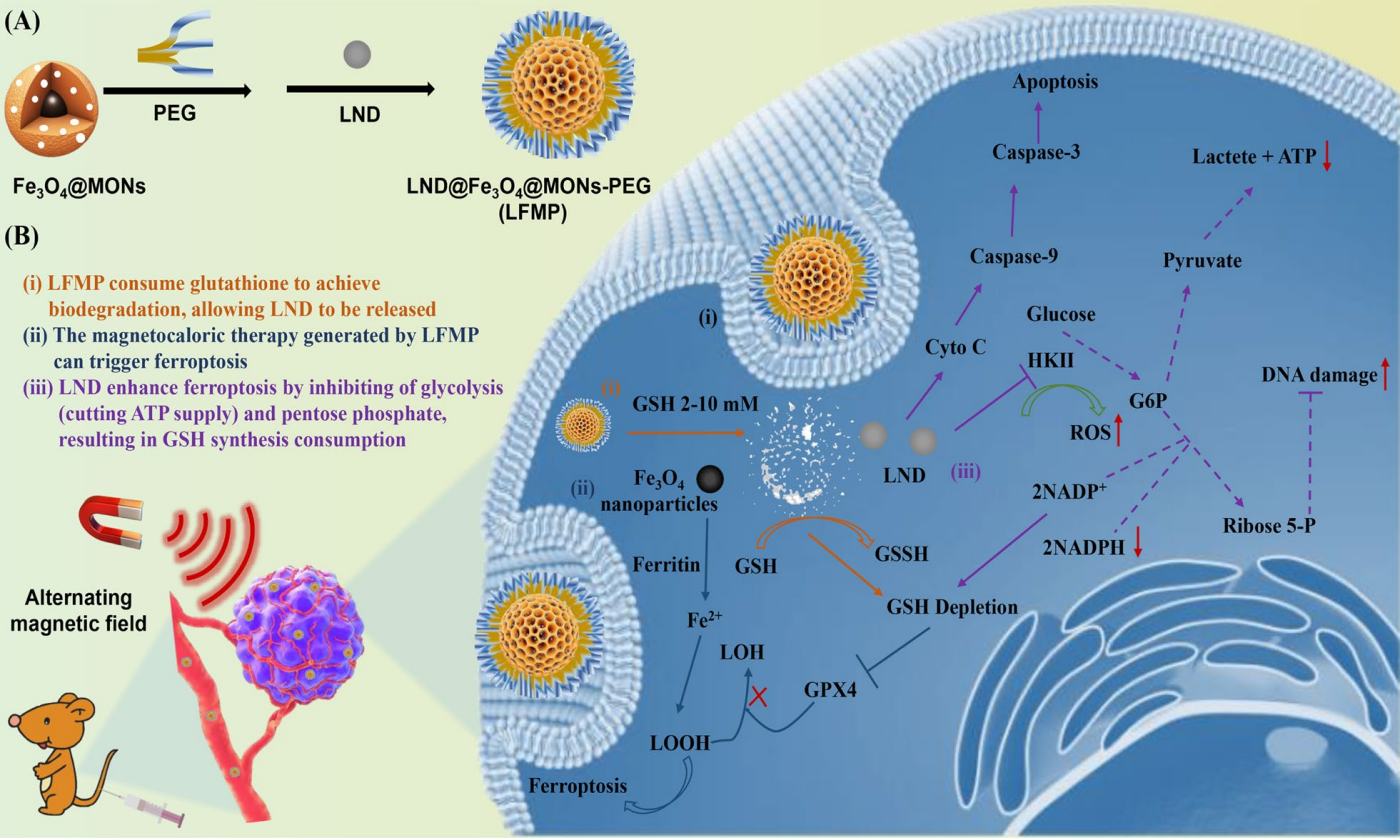
Schematic illustration composition of LND@Fe_3_O_4_@MONs-PEG (LFMP) and the therapeutic mechanism of LFMP. **A** Displaying the preparation process and nanostructure of LFMP. **B** The GSH responsive behavior of LFMP and its multiple anti-tumor effects by disrupting redox homeostasis, interfering with tumor cell metabolism, and triggering ferroptosis

In this study, we propose a ferroptosis-inducing nanoplatform with loaded LND and Fe_3_O_4_ based on a magnetic nanoparticle, characterized by exceptional thermal conversion properties and relaxation rates. The PEG-modified magnetic organic mesoporous silica nanoparticles (MONs-PEG), conjugated with bis[3-(triethoxysilyl)propyl]tetrasulfide (BTES) containing disulfide bonds as the organosilica precursor, were designed using conventional synthesis of magnetic mesoporous silica to deliver LND and Fe_3_O_4_ (Scheme 1). Following LFMP administration, the disulfide bonds, sensitive to GSH, underwent a sulfhydryl-disulfide exchange reaction within the nanoplatform, depleting GSH and inactivating GPX4. Upon the rupture of disulfide bonds, the nanoparticle decomposes and releases LND catalyzed by an alternating magnetic field (AMF) and a high level of GSH. Furthermore, LFMP induces a magnetic hyperthermia effect under an AMF. Consequently, the impairment of redox homeostasis and the accumulation of lipid peroxides are induced by the simultaneous disordered antioxidant system and enhanced ROS levels in tumor cells. LFMP was demonstrated to restrain glycolysis by reducing production of GSH, thereby inhibiting the proliferation of tumor cells. Overall, this study innovatively reveals that LFMP effectively promotes ferroptosis by impairing redox and iron homeostasis, leading to accumulated ROS and depleted GSH in tumor cells. These results demonstrate the feasibility of using Fe_3_O_4_-based nanodrugs to induce tumor ferroptosis and enhance ferroptosis through glucose metabolism intervention. T

## Materials and methods

### Materials and reagents

Bis(triethoxysilylpropyl) disulfide (BTES), Cetyltrimethylammonium chloride (CTAC), *N*-hydroxysuccinimide (NHS, > 97.0%), Tetraethylorthosilicate (TEOS), *N*-(3-Dimethylaminopropyl)-N0-ethylcarbodiimide hydrochloride (EDC, > 98.0%), triethanolamine (TEA), 3-aminopropyltriethoxysilane (APTES), LND and were obtained from Sigma-Aldrich (MO, USA). All the chemicals were used as received without further purification. GSH assay kit, nti-SLC7A11 polyclonal antibody, and 2′,7′-dichlorofluorescein-diacetate (DCFH-DA) were purchased from Beijing Solarbio Science & Technology Co., Ltd. 4′,6-diamidino-2-phenylindole dihydrochloride (DAPI), Alexa Fluor 568 phalloidin, RPMI 1640 cell culture medium, and fetal bovine serum (FBS) were purchased from Gibco (Thermo Fisher Scientific, Waltham, MA, USA). Propidium iodide (PI), the cell counting kit (CCK-8), Calcein-AM, JC-1 probe, and ATP assay kit were obtained from the Beyotime Institute of Biotechnology (Shanghai, China). Ethanol was obtained from Kelong Chemicals (Chengdu, China). Anti-ACSL4 polyclonal antibody, anti-SLC3A2 polyclonal antibody, anti-GPX 4 polyclonal antibodies were obtained from Cell Signaling Technology (Beverly, MA, USA). The EMT-6 cells were obtained from the American Type Culture Collection (Manassas, VA, USA).

### Preparation of Fe_3_O_4_@MONs (FM) nanoparticles

The soft-templating methodology with minor modification was performed for synthesizing MON as previously published process [34]. Firstly, 200 μL Fe_3_O_4_ aqueous solution (10 mg/mL), 2 g CTAC and 0.08 g TEA was added into 20 mL deionized water for homogeneous mixing at 95 ℃ for 1 h. Then, 1 mL TEOS and 0.2 mL BTES were combined by ultrasonic and added to the aforesaid homogenous mixture, which was constantly stirred for 8 h under the same conditions. Finally, the MONs was extracted with 8 mg/mL sodium chloride (NaCl, dissolved in methanol) after being stirred at 40 °C for 48 h and repeated three times to remove the surfactants CTAC. Furthermore, the aminated nanoparticles (MONs-NH_2_) were produced using the traditional APTES methodology [35].

### Synthesis of Fe_***3***_O_4_@MONs-PEG (FMP) nanoparticles

An amide reaction was used to covalently modify the PEG layer on the MONs-NH_2_ surface. Briefly, the carboxyl group of PEG2000 (HOOC-CH2-O-(CH2CH2O-)-n-CH2-COOH) was dissolved by EDC and NHS in dimethyl sulfoxide (DMSO) solution at room temperature, and then added with the MONs-NH_2_ nanoparticles suspension for being continuously stirred at 25 ℃ for 48 h to obtain the reaction mixture the surface PEGylated Fe_3_O_4_@MONs (namely FMP), which was collected by 5 kDa molecular ultrafiltration tube. Finally, the purified solution was preserved through freeze-drying for further use.

### Synthesis of LND@Fe_***3***_O_4_@MONs-PEG (LFMP) nanodrug

For LND drug loading, the Fe_3_O_4_@MONs-PEG (50 mg) was dispersed into 50 mL of PBS (mixing with equal volume of DMF) containing 20 mg of LND stirred at room temperature. After stirring overnight, the loaded nanodrug particles (LND@Fe_3_O_4_@MONs-PEG named as LFMP) was fully dialyzed against purified water for 2 days, followed by centrifugation to remove unloaded LND.

### Drug loading and release of LFMP

In the process of LND@Fe_3_O_4_@MONs-PEG synthesis, the drug loading efficiency (LE) and drug encapsulation efficiency (EE) of LND were detected by high performance liquid chromatography (HPLC). The LE and EE were calculated using the following equations:

$${\text{LE}}=\frac{[{W}_{(total\ drug)}-{\text{W}}_{(drug\ in \ supernatant)}]}{W_{(nanoparticles)}}\times 100\%$$

$${\text{EE}}=\frac{{W}_{(drug \ in\  the\  nanoparticles)}}{W_{(total\ drug)}}\times 100\%$$

To further assess in vitro release behavior of LFMP, the dialysis bag diffusion method was used to assess the in vitro drug release profile of the LFMP. Specifically, LFMP (50 mg) was encapsulated into dialysis tubes (molecular weight cut off of 5000) which then was put in 50 mL PBS solutions at different GSH concentrations ([GSH] = 0, 5 and 10 mM). The amounts of LND released at different time points were measured by HPLC.

### Degradation behavior of FM, FMP and LFMP

To evaluate the GSH-responsive disintegration characteristics of FM, FMP and LFMP, the biodegradation behavior of nanoparticles was studied in GSH solution. Typically, Fe_3_O_4_, FM and LFMP (5 mg/mL) were incubated with reductive PBS (pH 7.4, [GSH] = 5 mM) under stirring at 37 °C respectively. At predetermined time points (0, 2, 4, 8, 16, 32, and 72 h), 1 mL of solution was extracted and centrifuged to obtain the supernatant. Similarly, the concentration dependence of the FMP on GSH degradation was also investigated. FMP (0, 5, 10, 25, 50, 100 μg/mL) were incubated with reductive PBS (pH 7.4, [GSH] = 5 mM) under stirring at 37 °C for 24 h, respectively. Then, 1 mL of solution was extracted and centrifuged to obtain the supernatant. The GSH concentrations in these supernatants were detected using a GSH assay kit. For the intracellular GSH measurement, after the cells were treated with various formulations for 24 h, the supernatant was lysed and centrifuged to collect the supernatant, and the GSH concentration in the supernatant was then quantified using a GSH assay kit.

Also, LFMP (5 mg/mL) were incubated with reductive PBS (pH 7.4, [GSH] = 0, 5 and 10 mM) under stirring at 37 °C, respectively. Then, the average dynamic light scatterings of LFMP were measured after 24 h of co-incubation using a Zetasizer Nanoseries. The particle size and Zeta potential of LFMP in different culture media (PBS, saline, 1640 medium, fetal bovine serum and complete medium) was also detected.

### Magnetic thermal effect and thermal stability of LFMP

Different concentration (0, 50, 100, 200, 400 μg/mL) of LFMP aqueous solutions were exposed to AMF (17.5 kA/m, 250 kHz) for 600 s, and the temperature was recorded every 30 s. Then, the thermal stability of the LFMP solution was also detected, briefly, the LFMP solution (0.4 mg/mL) was placed in AMF (17.5 kA/m, 250 kHz) for 300 s and then naturally cooled in 10 min, the above procedures were repeated five times.

### Cell lines and cell culture

EMT-6 cells were provided by American Type Culture Collection. At 37 °C in a 5% CO2 environment, the cells were grown in RPMI1640 media containing 10% fetal bovine serum (FBS), penicillin (100 U/mL), and streptomycin (100 U/mL). And for all experiments, 0.25% trypsin–EDTA was used to detach and collect cells, and the pelleted cells were resuspended in a fresh medium before the subsequent experiment.

### Cytotoxicity assays

The biosafety of Fe_3_O_4_, MP and LND synthesized in this research were assessed in vitro by a CCK-8. Briefly, EMT-6 cells were seeded into 96-well plates (4 × 10^4^ cells per well) and cultured overnight. Then different concentrations of Fe_3_O_4_ (0, 10, 50, 100, 150, 200, 250 μg/mL), MP (0, 20, 40, 80, 160, 200, 400 μg/mL) and LND (0, 25, 50, 100, 150, 200, 250 μg/mL) in culture medium were respectively added into the wells and co-incubated for 24, 48, 72 h. Then the old medium was removed and all the groups were treated with the CCK-8 assay following the manufacture’s protocol. The antitumor effect in vitro was also studied by evaluating the viability of the EMT-6cancer cells by a CCK-8 assay after treating with FMP, LFMP, FMP + AMF and LFMP + AMF at an equivalent concentration of LND for 24 h. After 8 h of treatment, the AMF groups were treated with alternative magnetic field (17.5 kA/m, 250 kHz) for 10 min, and the contrast group was protected from AMF. Unless otherwise stated, the AMF used in cell experiments below are consistent with the description above.

Except for the CCK-8 assay, live/dead cell staining assay was also used to evaluate cytotoxicity via CLSM. Specifically, EMT-6 cells seeded in a six-well plate (4 × 10^5^ cells per well) were treated with FMP, LFMP, FMP + AMF and LFMP + AMF (150 μg/mL of LND equivalent) for 24 h. After incubation, Calcein-AM (1 μL)/propidium iodide (PI, 3 μL) were added to the medium and incubated for 30 min. Finally, the cells were washed with PBS (pH 7.4) three times and imaged by CLSM. The red and green fluorescence analysis of fluorescence imaging was completed by ImageJ software.

### Determination of intracellular ROS

The ROS generation ability of LND was evaluated by CLSM and FACS with a DCFH-DA probe. EMT-6 cells were seeded into 6-well plates at a density of 4 × 10^5^ per well overnight. Subsequently, the culture medium was replaced with fresh one containing the LND (50, 100, 200, 400 μg/mL), FMP, LFMP, FMP + AMF or LFMP + AMF at an equivalent concentration ([LND] = 150 μg/mL) for another 24 h incubation. The cells after various treatments were stained with the DCFH-DA fluorescent probe according to the manufacturer’s protocol and observed via CLSM (λ_ex_: 488 nm, λ_em_: 525 nm).

### Mitochondrial membrane depolarization

Mitochondrial membrane potential (ΔΨm) was assessed according to the manufacturer’s instruction with a mitochondrial membrane potential assay kit with a JC-1 (C2006, Beyotime, China). EMT-6 cells were treated with FMP, LFMP, FMP + AMF or LFMP + AMF at an equivalent concentration ([LND] = 150 μg/mL) for another 24 h incubation. Then, cells were treated with JC-1 fluorescent probe at 37 ℃ for 30 min. The stained cells were washed twice and observed by CLSM. Mitochondrial depolarization was indicated by cells that shift from red to green fluorescence.

### Determination of intracellular MDA levels

EMT-6 cells were seeded into 6-well plates at a density of 4 × 10^5^ per well overnight. Subsequently, the culture medium was replaced with fresh medium containing FMP, LFMP, FMP + AMF or LFMP + AMF at an equivalent concentration ([LND] = 150 μg/mL) for another 24 h incubation, respectively. Then the intracellular MDA levels were investigated by lipid peroxidation MDA assay kit (Beyotime, China) the intracellular MDA according to the operating instructions.

### Intracellular Lipid peroxide level assessment

To visually observed the lipid peroxide levels in EMT-6 cells, cells were seeded into 6-well plates at a density of 4 × 10^5^ per well overnight. Subsequently, the culture medium was replaced with fresh medium containing FMP, LFMP, FMP + AMF or LFMP + AMF at an equivalent concentration ([LND] = 150 μg/mL) for another 24 h incubation, respectively. After that, BODIPY-C11 (5 μM) as a lipid peroxide probe was added to 30 min. The fluorescence imaging of intracellular LPO was observed via CLSM (λ_ex_: 581 nm, λ_em_: 591 nm).

### Western blot analysis

Expression of the GPX4 proteins in EMT-6 cells was detected by Western blot. Briefly, EMT-6 cells were seeded in 6-well plates and incubated with Fe_3_O_4_, FMP, LFMP, FMP + AMF or LFMP + AMF at an equivalent concentration ([Fe] = 0.6 mM) for another 24 h incubation, respectively. The cells without any treatment served as control. Then, cells were lysed in RIPA lysis buffer (Beyotime, China), and the protein concentration was detected by the BCA protein assay kit (Beyotime, China). Subsequently, various protein groups were isolated by 12% SDS-PAGE and moved to PVDF membranes (Millipore, Bedford, MA, USA). PVDF membranes were blocked at room temperature for 3 h in Tris Buffered Saline Tween (TBST) buffer and incubated with the primary antibodies (GPX4, Beyotime, China) at 4 °C overnight. After the membranes were washed to eliminate non-bound primary antibodies, the membranes were incubated with the corresponding secondary antibodies at a 1: 5000 dilutions at room temperature for 2 h. The membranes were washed three times with TBST, and the chemiluminescence and fluorescence imaging system observed immunoreactive signals (Sagecreation, China). ImageJ software was used to quantify the various protein expression.

### Monitoring intracellular ATP content

EMT-6 cells were seeded into 6-well plates at a density of 4 × 105 per well overnight. Subsequently, the culture medium was replaced with fresh one containing FMP, LFMP, FMP + AMF or LFMP + AMF at an equivalent concentration ([LND] = 150 μg/mL) for another 24 h incubation. Then the cells were followed by lysis on ice and centrifugation (12000*g*, 5 min) at 4 °C. The supernatants were quantified using an ATP assay kit (Beyotime, Shanghai).

### Detection of intracellular/extracellular lactate content

EMT-6 cells were seeded into 6-well plates at a density of 4 × 105 per well overnight. Subsequently, the culture medium was replaced with fresh medium containing FMP, LFMP, FMP + AMF or LFMP + AMF at an equivalent concentration ([LND] = 150 μg/mL) for another 8, 12, 16, 20, 24, 36 and 48 h incubation, respectively. Then the medium and cells was collected at 8, 12, 16, 20, 24, 36 and 48 h. After that the extracellular and the intracellular lactate content of the different groups was measured using a lactate kit (Nanjing Jiancheng Institute of Biological Engineering) following the manufacture’s protocol. The protein content of the EMT-6 cells was determined in each group by a BCA kit (Nanjing Jiancheng Institute of Biological Engineering). Finally, the lactate content units were converted to mmol/g protein.

### Animals and tumor model

BALB/c female mice (6–8 weeks old) were purchased from Chongqing Ensiweier Biotechnology Co., Ltd. (Chongqing, China). Animals were subsequently treated after EMT-6 cell inoculation when the tumors reached a volume of approximately 100 mm^3^ (volume = length × width^2^/2). All animal procedures were in accordance with the specifications of the Guide for the Care and Use of Laboratory. Animals, all experiments were performed following the protocol approved by the Institutional Animal Care Committee of the University of Electronic Science and Technology of China (UESTC).

### In vivo MR imaging

To assess biodistributions of nanocomplexes, Fe_3_O_4_ and LFMP to be used as a MR imaging agent to visualize the tumor accumulation on EMT-6 tumor-bearing mice ([Fe] = 2 mg/kg). At various time points (0, 8, 24 and 48 h) post i.v injection, MR *T2*-weighted imaging on each mouse using a special coil. The parameters are as follows: TR = 2000 ms, TE = 81.9 ms, FOV = 12 × 12 cm, image matrix = 256 × 160, slice thickness = 2 mm.

### In vivo antitumor effects

EMT-6 tumor-bearing mice were grouped at random and intratumorally injected with saline, LND, FMP + AMF, or LFMP + AMF (4 mg/kg of LND equivalent, AMF: 17.5 kA/m, 250 kHz). FMP + AMF and LFMP + AMF groups containing AMF were treated with AMF for 10 min. The above treatment plan is repeated every other day, and a total of 3 treatments are given throughout the entire treatment period. During the treatment process, body weight and tumor volume were recorded every three days. At the end of treatment, the tumors and blood sample were harvested for subsequent analysis. The blood sample withdrawn from the mice for blood chemistry tests and routine blood analysis. To further verify the therapeutic effect of nanoplatform, ki67 and TUNEL assays of tumor sections were conducted. For ferroptosis evaluation, immune-fluorescence analysis of tumor slices was performed to estimate the expression of GPX4, ACSL4, SLC3A2, and SLC7A11.

### Characterizations

The morphology of FMP and LFMP were observed by TEM. The particle size and zeta potential were detected by dynamic light scattering (DLS) analysis (Zetasizer Nano ZS90, Malvern, UK). Scanning electron microscope (SEM) images, element mapping images, and elements line scanning results were obtained on a field-emission Magellan 400 microscope (FEI Co.). The hemolysis rate of the LFMP were conducted on a UV–visible spectrometer (UV-2910, Hitachi, Japan). The pore size and specific surface area of the nanoparticles were determined by Brunauer–Emmett–Teller (BET) analyzer (Micromeritics, ASAP 2020). The drug loading efficiency (LE) and drug encapsulation efficiency (EE) of LND were detected by HPLC.

### Statistical analysis

The statistical calculations of the results were carried out by Prism 6.0 software (GraphPad Software Inc., San Diego, CA, USA) and all data were expressed as mean ± standard derivations (SD). Multigroup analyses were made by one-way analysis of variance (ANOVA) followed by a Student t-test and p values below 0.05 were deemed statistically important *p < 0.05, **p < 0.01, and ***p < 0.001.

## Results and discussion

### Construction and characterization of the LFMP

**Fig. 1:**
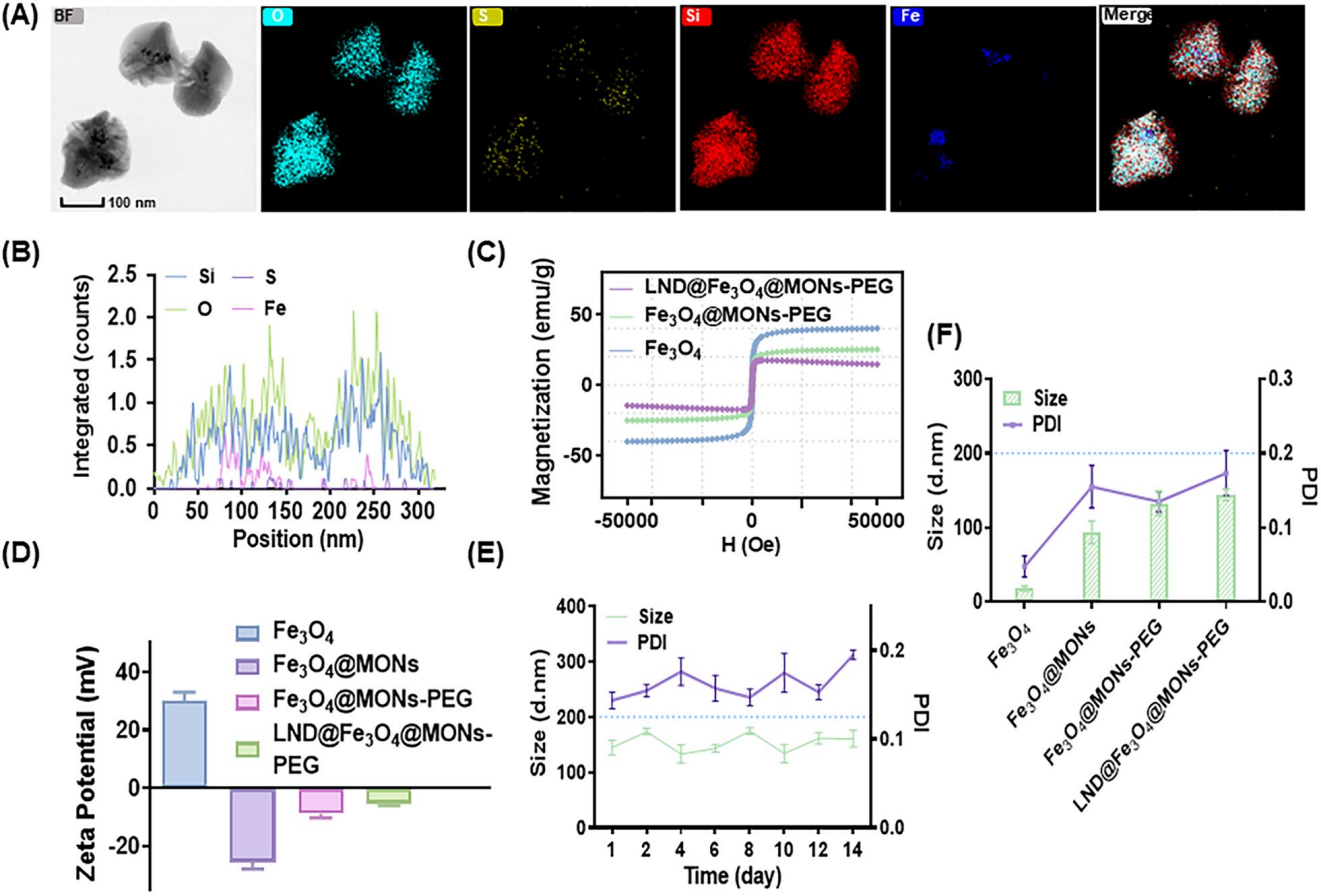
**A** Representative TEM images and elemental mappings of O, S, Si and Fe elements for Fe_3_O_4_@MONs. **B** Linear elemental scanning of Fe_3_O_4_@MONs. **C** Field-dependent magnetization curves of Fe_3_O_4_, Fe_3_O_4_@MONs-PEG, and LND@Fe_3_O_4_@MONs-PEG at room temperature. **D** Zeta potential of Fe_3_O_4_, Fe_3_O_4_@MONs, Fe_3_O_4_@MONs-PEG and LND@Fe_3_O_4_@MONs-PEG. **E** Hydrodynamic diameter distributions and corresponding PDI of LND@ Fe_3_O_4_@MONs-PEG dispersed in PBS (pH = 7.4) during 14 days. **F** Hydrodynamic diameter distributions and corresponding PDI of Fe_3_O_4_, Fe_3_O_4_@MONs, Fe_3_O_4_@MONs-PEG and LND@Fe_3_O_4_@MONs-PEG dispersed in PBS (pH = 7.4). Data are shown mean ± SD, n = 5

In Scheme 1A, a GSH-responsive mesoporous silica nanodrug with a Fe_3_O_4_ core–shell structure was designed for delivering LND, denoted as LFMP, in antitumor therapy. Briefly, Fe_3_O_4_ was designed as the core of MONs with a skeleton of disulfide bridges. LND was then loaded into the PEG-modified MONs structure to form LFMP, a nanomedicine that released in response to GSH. Following intra-tumoral injection into MET-6-tumor-bearing mice, LND was released in the tumor microenvironment (TME) in response to the high concentration of GSH. This release was expected to enhance ferroptosis by inhibiting glycolysis (cutting ATP supply) and the pentose phosphate pathway, resulting in GSH synthesis consumption. Under the influence of an AMF, Fe_3_O_4_ could generate synergistic magnetothermal anti-tumor therapy (Scheme 1B). As shown in Fig. 1A, the topographical features of FM nanoparticles were characterized via transmission electron microscopy (TEM), and elemental maps of Fe, O, Si, and S confirmed the composition consistent with the prospective design. This demonstrated the successful modification of Fe_3_O_4_ in the core of FM and the presence of disulfide bond groups on the surface of FM. Moreover, to visualize the insertion of an organic group, typical spherical structures such as –S–S– were checked for component distributions by elemental line scanning (Fig. 1B). After a series of modifications and drug-loading, LFMP still showed a uniform spherical dispersion and with an average diameter of 160.85 ± 22.93 nm Additional file 1: Fig. S1). The magnetization results showed that LFMP exhibited superparamagnetic behavior, with a saturation magnetization up to 17.437 emu/g, suggesting that the silicon shell encapsulation and surface modification of Fe_3_O_4_ did not affect their functionality (Fig. 1C). Additionally, the capability of the nanoplatform as an MR imaging contrast agent was investigated using a clinical 3.0 T MR imaging device. Significantly, as the concentration of Fe in LFMP nanoparticles increased, the MRI imaging dimmed (Additional file 1: Fig. S2A), the *r*^*2*^ value reached up to 62.91 mM − 1 s − 1 (Additional file 1: Fig. S2B), and the signal intensity decreased (Additional file 1: Fig. S2C), while the effect of *T2*-weighted imaging was better. These results clearly verified the potential of LFMP as a *T2*-weighted MR imaging contrast agent for tumors.

Additionally, the zeta potentials of Fe_3_O_4_, FM, FMP and LFMP were determined to be 30.19 ± 2.63, − 25.64 ± 2.05, − 8.87 ± 1.32 and − 5.35 ± 0.68 mV, respectively (Fig. 1D). This indicated a significant charge reversal during the synthesis process of nanoparticles, indicating smooth drug loading and modification. N_2_ adsorption desorption isotherms and related pore-size distributions of nanoparticles are shown in Additional file 1: Table S1. The mesoporous structures of FM and LFMP were measured by the type IV isotherm and the corresponding pore size distributions, revealing a decrease in surface area from 626.47 m^2^/g to 356.72 m^2^/g, and a marginal decrease in pore volumes from 0.64 cm^3^/g to 0.13 cm^3^/g, respectively. Consequently, the pore volume remained sufficient for LND to be loaded into FMP after functionalization. No significant variations in size and polydispersity (PDI) of LFMP were observed after incubated in PBS for 14 days (Fig. 1E). DLS measurements confirmed that the LFMP (5 mg/mL) hydrodynamic diameter and zeta potential changed slightly when dispersed in different mediums, demonstrating the remarkable stability of nanoparticles (Additional file 1: Table S2).

Additionally, the average particle size gradually increased with further modification (Fig. 1F), and the average particle size of LFMP (144.34 ± 7.63 nm) was larger than that of Fe_3_O_4_ (18.18 ± 3.3 nm), FM (93.78 ± 15.31 nm), and FMP (132.91 ± 16.64 nm). Overall, the observations proved that the LFMP synthesized in this study exhibited a core–shell structure, and its appropriate particle size can facilitate accumulation at the tumor site through the enhanced permeability and retention (EPR) effect. Following incubating with different concentration of LFMP, 0.9% NaCl, and water over 4 h respectively, no indication of hemolysis was found in LFMP and 0.9% NaCl group (Additional file 1: Fig. S3), suggesting that FM was stabilized by PEG in serum and that this system is suitable for in vivo application.

### Responsive release study, GSH depletion capacity assay and magnetic property of LFMP in vitro

**Fig. 2:**
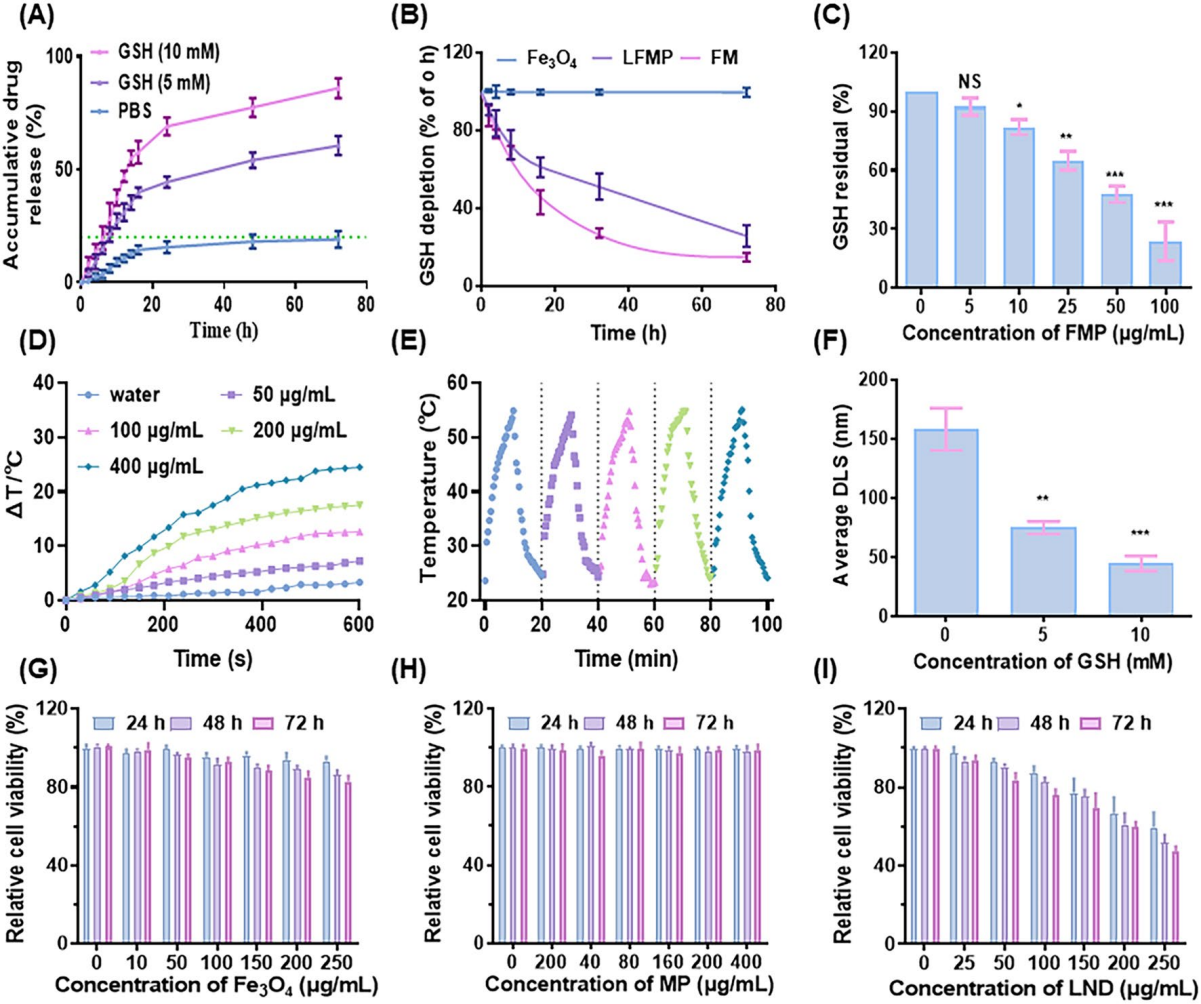
**A** Release profiles of LND from LFMP with or without GSH. **B** The change of GSH level of Fe_3_O_4_, FM, and LFMP in PBS solution ([GSH] = 5 mM) during 72 h. **C** The ability of FMP to consume GSH ([GSH] = 5 mM) under different concentration conditions. **D** Quantitative temperature curve of LFMP in vitro. **E** Heating curves of the LFMP (0.4 mg/mL) exposed to the AMF (17.5 kA/m, 250 kHz) for five on/off cycles. **F** Corresponding average dynamic light scatterings (DLS) diameter of LFMP in GSH. **G** Viabilities of EMT-6 cells co-incubated with different concentrations of Fe_3_O_4_ for 24 h, 48 h, 72 h. **H** Viabilities of EMT-6 cells co-incubated with different concentrations of MP for 24 h, 48 h, 72 h. **I** Viabilities of EMT-6 cells co-incubated with different concentrations of LND for 24 h, 48 h, 72 h. Data are shown mean ± SD, n = 5. *p < 0.05, **p < 0.01, ***p < 0.005

Given that the LFMP containing a disulfide bond, could oxidize GSH to GSSG via a mercaptan disulfide bond exchange reaction, we assessed the LFMP drug release within a set time interval after treating it with different concentrations of GSH. Initially, we determined the drug loading efficiency (23.25%) and drug encapsulation efficiency (83.01%) of LND during the drug synthesis process using HPLC. Additional studies will be necessary to further confirm that the nanodrug designed in this study can achieve effective drug delivery and responsive drug release. As illustrated in Fig. 2A, approximately 45.18% and 60.73% of LND were released from LFMP when incubated in PBS ([GSH] = 5 mM) within 24 or 72 h, respectively. In contrast, only 18.25% of LND was detected from LFMP after 72 h of incubation when LFMP were dispersed in the solution without GSH. Specifically, less than 20% of LND leaked from the nanodrug after 72 h of incubation without a GSH solution, once again indicating the good stability of LFMP. This suggested that FMP could prevent the leakage of LND when the particles circulate in the blood. In contrast, LFMP in the 10 mM GSH solution showed a rapid increase in the released value, reaching 72.83% after 24 h. The underlying rationale was that the disulfide bond in the particle can be broken in an environment where GSH exists [36, 37], leading to the rapid release of LND. The representative TEM images of biodegradation behavior of LFMP in PBS solution ([GSH] = 10 mM) for 10 days are presented in Additional file 1: Fig. S4.

To assess the GSH depletion capability of the nanodrug, Fe_3_O_4_, FM, and LFMP were incubated in a PBS buffer containing GSH (5 mM) to simulate the reducing tumor microenvironment (TME). The time-dependent study revealed a gradual decrease in GSH concentration over time, attributed to the abundance of disulfide bonds in LFMP, with 75.64% of GSH being depleted within 24 h (Fig. 2B). Fe_3_O_4_, serving as a negative control, did not influence the concentration of GSH in the environment due to the absence of disulfide bonds. Additionally, as the amount of FMP gradually increased, the residual amount of GSH also decreased progressively. When the FMP concentration reached 100 μg/mL, only 23.85 ± 9.79% of GSH remained (Fig. 2C). The reduction in particle size observed after LFMP incubation with GSH served as evidence for the GSH-responsive degradation of the nanodrug (Fig. 2F). To better assess the performance of the nanodrug, we comprehensively evaluated its magnetocaloric effects under the influence of an AMF. The temperature increased in a concentration-dependent manner and stabilized after 600 s of treatment (Fig. 2D). At an LFMP concentration of 400 μg/mL, the temperature rise difference under AMF reached 25.3 ℃. Moreover, the temperature consistently increased to 54.1–55.1 ℃ during all five on/off cycles of the AMF (Fig. 2E), demonstrating the excellent thermal conversion efficiency and stability of LFMP.

### Evaluation on cellular toxicity

**Fig. 3:**
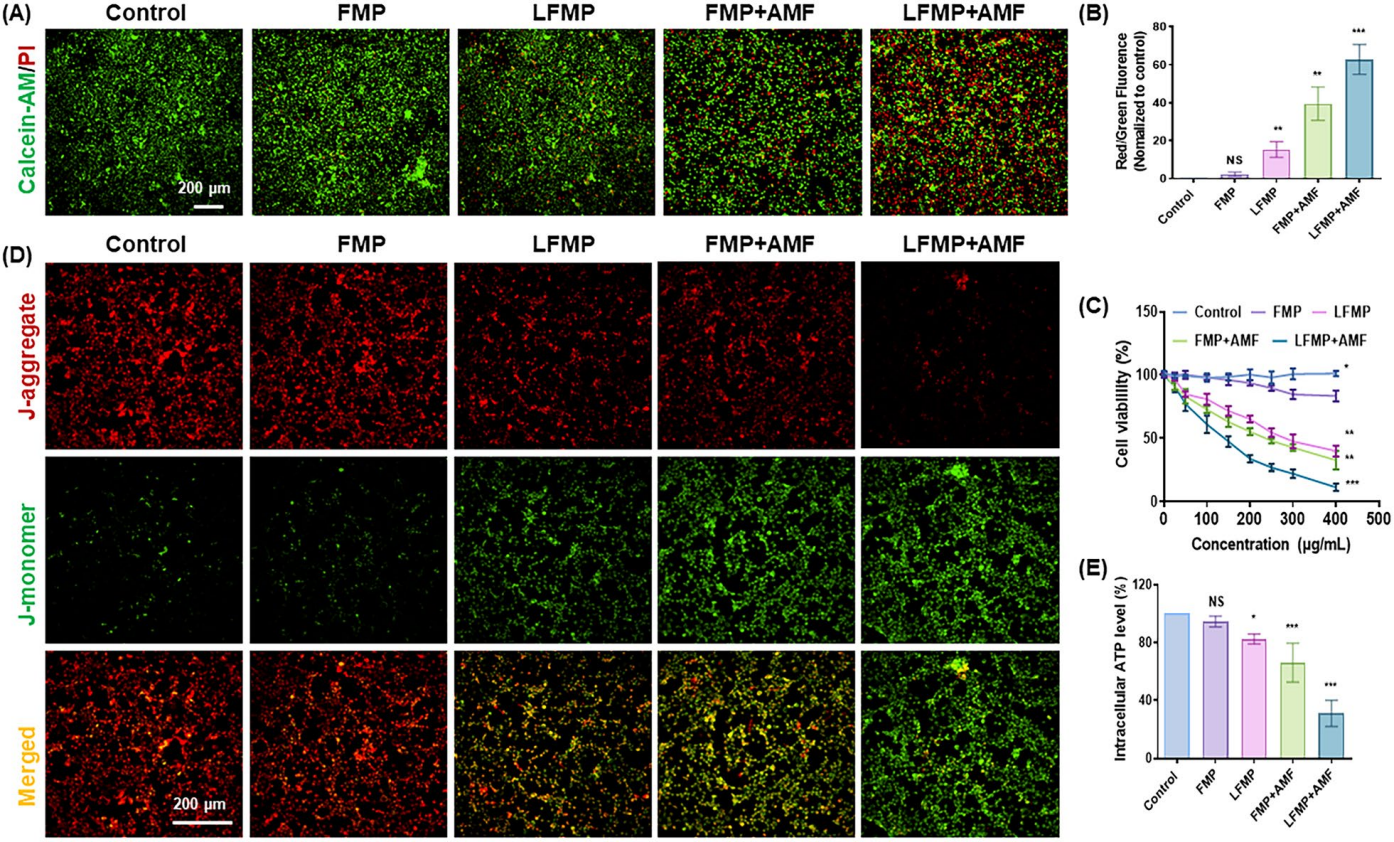
**A** Live/dead cell staining assay of the EMT-6 cells after treatment with different groups (scale bar = 200 µm). **B** Statistical analysis of red/green fluorescence in **A** by flow cytometry. **C** The cytotoxicity of EMT-6 cells with different treatments. **D** Determination of the population of polarized/depolarized mitochondria using JC-1. In apoptotic cells, JC-1 exists in the monomeric form because of the low mitochondrial membrane potential, staining the cytosol green. In live nonapoptotic cells, JC-1 accumulates as aggregates in the mitochondrial membrane which stains red. **E** Concentrations of intracellular ATP using an ATP Determination Kit. Data are shown mean ± SD, n = 5. *p < 0.05, **p < 0.01, ***p < 0.005

Encouraged by the magnetic properties of LFMP in vitro, the synergistic therapeutic performance of LFMP was systematically evaluated. It is widely recognized that low toxicity is essential for future applications as a biological nanomaterial and medication carrier [38, 39]. Therefore, the toxicity of LFMP was measured and analyzed through the CCK-8 experiment. Considering the negligible cytotoxicity of Fe_3_O_4_ (Fig. 2G), a corresponding concentration of Fe (2 mg/kg) was applied to evaluate the MR imaging of Fe_3_O_4_ and LFMP. The cell viability of EMT-6 cells treated with MP (Fig. 2H) was maintained at more than 90% when the nanocarrier concentration exceeded 400 μg/mL. Following PEG modification, MP exhibited improved biosafety to cells, attributed to PEG's ability to enhance nanoparticle biocompatibility. Notably, the low concentration of LND (< 100 μg/mL) showed a negligible cytotoxic effect due to its poor solubility in the cytoplasm (Fig. 2I). Furthermore, the IC50 value of free LND was 276.2 μg/mL against EMT-6 cells (Additional file 1: Fig. S5A). Live/dead cell staining assays were performed to visually evaluate the synergistic anti-tumor effect under AMF and glucose metabolism intervention (Fig. 3A). LFMP + AMF induced almost complete cell death, as evidenced by the widespread red dots in the observation zone. The statistical results also confirmed this phenomenon (Fig. 3B).

As illustrated in Fig. 3C, FMP was ineffective in inhibiting cell growth even at a concentration of 400 μg/mL, potentially due to its excellent biocompatibility. In contrast, LFMP-treated cells exhibited a noticeable decrease in viability in an LND-dependent manner. Given the negligible cytotoxicity of free LND, the impact of LND in FMP might be attributed to the enhanced cellular uptake facilitated by LFMP. Moreover, FMP-mediated magnetothermal therapy demonstrated robust anti-tumor effects, reducing the viability of EMT-6 cells by 70.69% at a concentration as high as 400 μg/mL. In contrast, LFMP + AMF treatment resulted in the lowest cell survival rate reaching 8.27%, indicating a more effective collaborative therapy. Since FMP modification did not induce direct cytotoxicity, FMP had the highest IC50 values, measuring 1047 μg/mL (Additional file 1: Fig. S5B). Additionally, LFMP + AMF exhibited an IC50 value of 129.1 μg/mL, significantly higher than those of LND and PMVL, suggesting that LND loading contributed to boosting the cytotoxicity of FMP + AMF (Additional file 1: Fig. S5C). Furthermore, we sought to explore potential mechanisms through which LND could enhance the cell-killing effect of FMP + AMF, an inquiry addressed in the subsequent sections. To elucidate the synergistic anti-tumor effects of LND and FMP more comprehensively, we employed CompuSyn software for calculating the Combination Index (CI) and assessing the synergistic effects of the drug. CompuSyn is widely utilized for drug combination effect analysis, relying on the classic Chou-Talalay method. It computes the combination index by scrutinizing the dose–response curve of drugs. The calculated CI value is indicative of the synergistic effect of drug combinations: a CI value less than 1 signifies a synergistic effect, equal to 1 suggests an additive effect, and greater than 1 implies an antagonistic effect. As shown in Additional file 1: Fig. S6, after loading LND into FMP, the CI values of LFMP and LFMP + AMF were all < 1, demonstrating a strong synergistic effect.

### In vitro ferroptosis assay and glycolysis intervention-mediated ferroptosis

#### Sensitization

In line with the aforementioned findings, LFMP facilitated the release of LND in response to GSH and activated ferroptosis, potentially by inhibiting GSH levels and GPX4 expression. This mechanism could contribute to the apoptosis of tumor cells. Our hypothesis suggests that LFMP may exert synergistic anti-tumor efficiency by promoting ferroptosis and oxidative stress in tumor cells. Mitochondrial function is intricately linked to ROS generation, ferroptosis, and apoptosis processes [40–42]. To explore this further, we conducted a JC-1 fluorescent assay to assess mitochondrial membrane potential in EMT-6 cells. As illustrated in Fig. 3D, both LFMP and LFMP + AMF significantly reduced MMP, hastening the apoptosis of tumor cells with mitochondrial impairment. Additionally, tumor cell proliferation primarily relies on ATP generated from mitochondria, and impaired mitochondrial function exacerbates ATP depletion. Consistently, a reduction in ATP content was observed in tumor cells after LFMP + AMF administration (Fig. 3E). These results indicate that LFMP exerted a remarkable anti-tumor effect through the synergistic function of the GSH-responsive nanoparticle, possibly by disrupting mitochondrial function and ATP content, thereby inducing ferroptosis and enhancing tumor cell death.

**Fig. 4:**
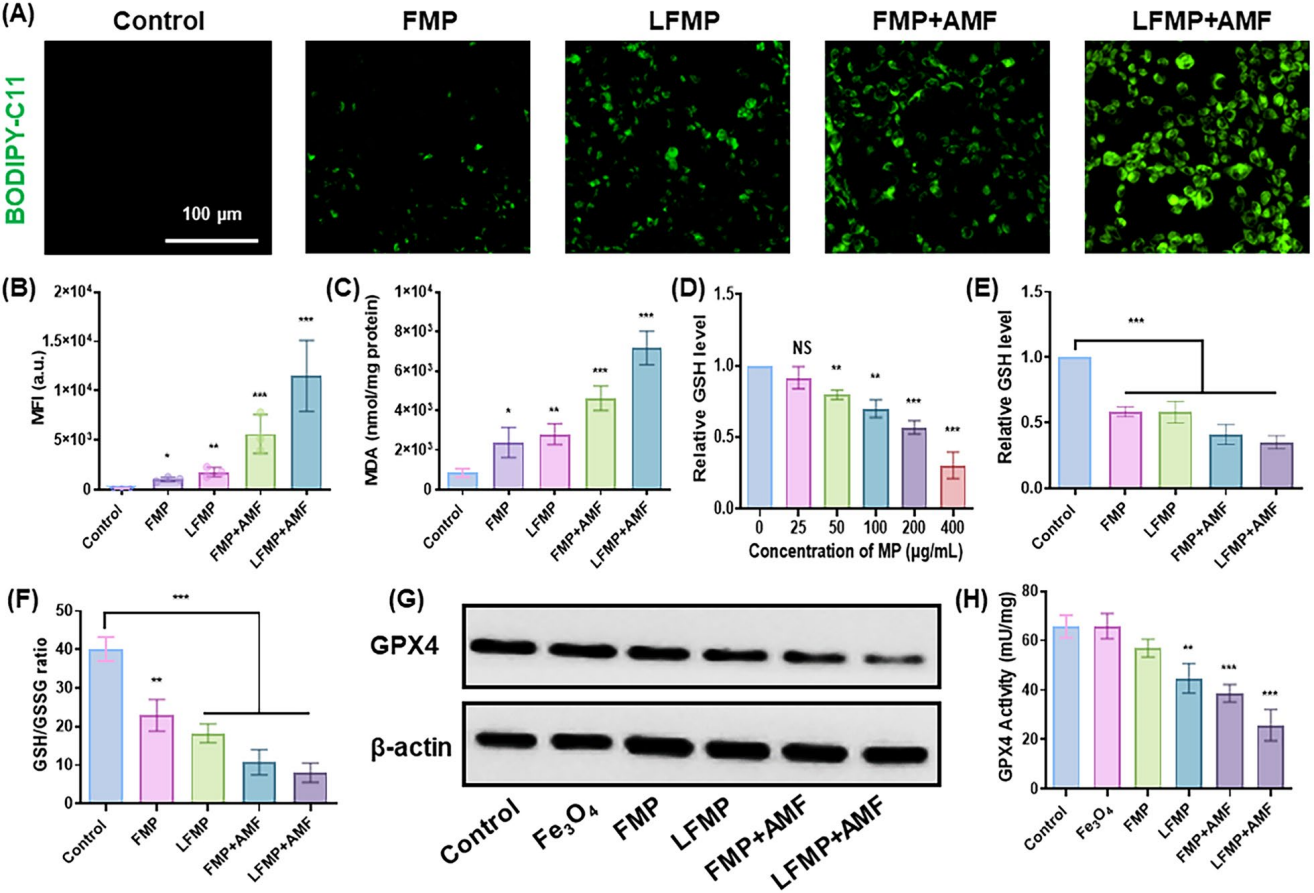
Detection of ferroptosis and disruption of dual homeostasis within tumor cells by LFMP. **A** Evaluation of lipid peroxidation in EMT-6 cells under different treatments with BODIPY-C11 (scale bar = 100 µm). **B** Lipid peroxidation was measured by flow cytometry with BODIPY-C11 staining. **C** Intracellular MDA levels of EMT-6 cells under different treatment therapy. **D** The relative GSH levels in EMT-6 cells treated with MP at different concentrations. **E** The relative GSH levels in EMT-6 cells were subjected to different treatments. **F** Glutathione (GSH)/oxidized glutathione (GSSG) ratio in EMT-6 cells under different treatment. **G** Assessment of GPX4 expression under different treatments. **H** Enzyme activity of GPX4 in EMT-6 cells with different treatments. Data are shown mean ± SD, n = 5. *p < 0.05, **p < 0.01, ***p < 0.005

GSH depletion was closely related to ROS accumulation, promoting the occurrence of ferroptosis. BODIPY-C11 staining, a classic indicator of ferroptosis [43], was used to detect ferroptosis in tumor cells. As depicted in Fig. 4A and B, the BODIPY-C11 marker with green fluorescence increased in the FMP and LFMP groups compared to the control group, with significantly higher green fluorescence in the LFMP group compared to the FMP group. Additionally, BODIPY-C11 fluorescence was further enhanced in the FMP + AMF and LFMP + AMF groups compared to the FMP and LFMP groups, respectively. This indicated higher levels of lipid peroxidation in tumor cells after LFMP or LFMP + AMF treatments. Propanondialdehyde (MDA), a typical product of ferroptosis-related lipid peroxidation, was monitored to estimate the degree of nanoparticle-induced ferroptosis in tumor cells [44]. As demonstrated in Fig. 4C, LFMP significantly promoted the production of intracellular MDA compared to other groups. Moreover, the relative GSH levels were declining in tumor cells after MP administration compared to others, and the decrease occurred in a concentration-dependent manner (Fig. 4D). The nanoparticles we designed significantly reduced GSH levels to varying degrees compared to the control (Fig. 4E). Similarly, the ratio of GSH/GSSG was lower in LFMP or LFMP + AMF groups compared to other groups (Fig. 4F).

It is well known that GPX4 was the enzyme responsible for clearing lipid peroxides in cells and plays a crucial role in maintaining the lipid bilayer homeostasis of cell membranes [45]. The inhibition of GPX4 could directly induce ferroptosis [46]. We observed a significant decline in GPX4 protein expressions after nanoparticle treatments, particularly in the LFMP or LFMP + AMF groups, compared to the control (Fig. 4G and H). GSH was considered a co-factor of GPX4, and GSH concentration was positively correlated with GPX4 expression [47]. Given the apparent reduction in GPX4 and GSH levels we determined in the LFMP treatment group, it implied that LFMP or LFMP + AMF could effectively promote ferroptosis by inhibiting GPX4 activity, thus demonstrating great potential in anti-tumor activity.

### In vitro synergistic anti-tumor effect of LFMP and the possible mechanism

**Fig. 5:**
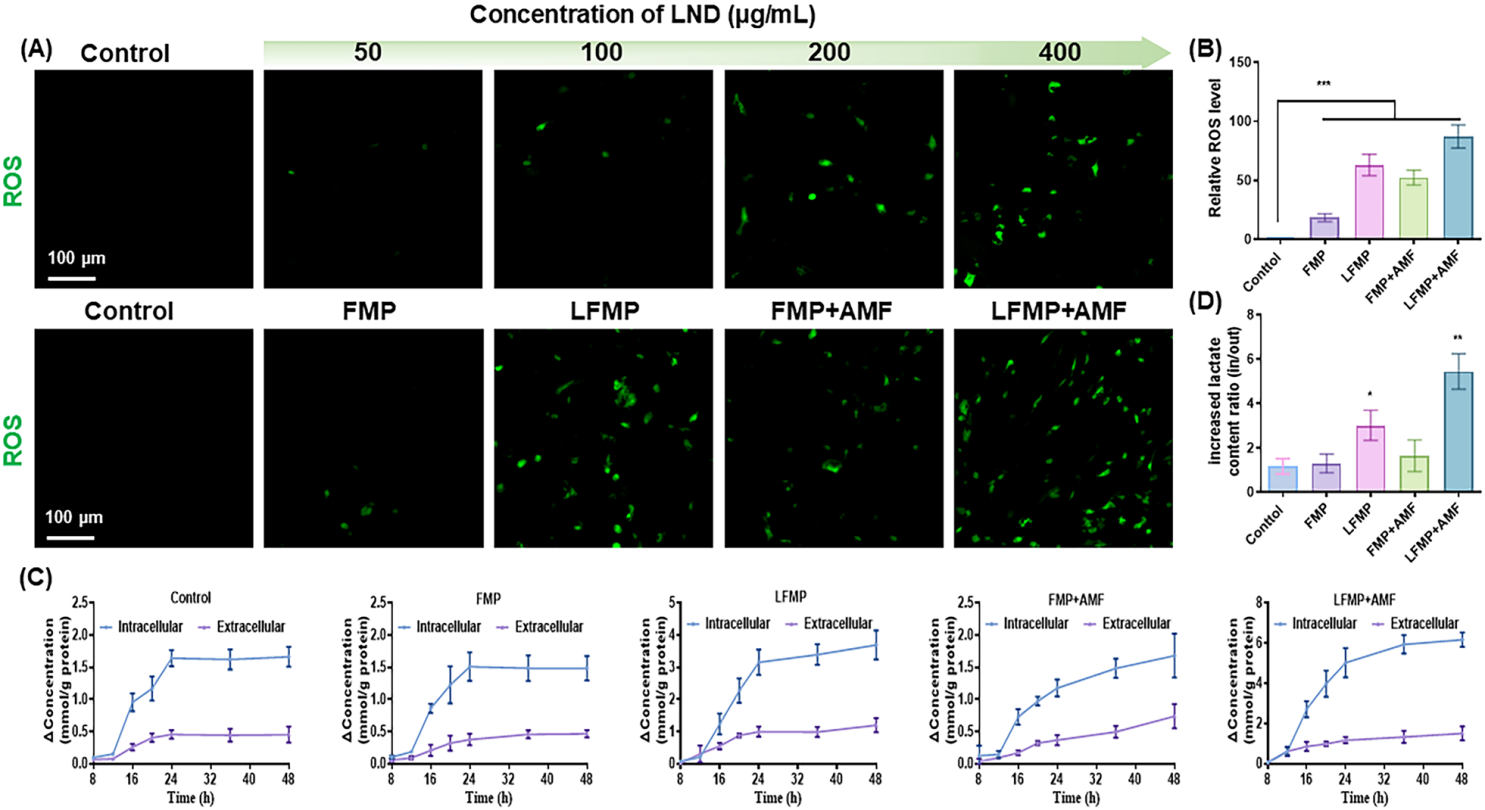
**A** Representative CLSM images of ROS produced by EMT-6 cells after different treatments (scale bar = 100 μm). **B** Flow cytometry analysis of cellular ROS generation after treated with FMP, LFMP, FMP + AMF, LFMP + AMF against EMT-6 cells. **C** The intracellular and extracellular increased content of lactic acid after being treated with different samples. **D** The ratio of intracellular and extracellular lactic acid increased content after being treated with different samples. Data are shown mean ± SD, n = 5. *p < 0.05, **p < 0.01, ***p < 0.005

The low antioxidant capacity results in the inability to scavenge ROS, contributing to ferroptosis therapy in some nanomedicine for tumors. We further assessed ROS levels in tumor cells through DCFH-DA staining. As shown in Fig. 5A, LND increased ROS generation in a concentration-dependent manner. LFMP or LFMP + AMF exhibited admirable effectiveness in promoting ROS accumulation (Fig. 5A and B). Considering that hypoxia was usually accompanied by lactate production and further accumulation in tumor tissue [48], we next focused on exploring the cascading effect of LFMP on metabolic regulation in tumor cells. In this study, the content of intracellular and extracellular lactate varied separately with different treatments.

As illustrated in Fig. 5C, the intracellular lactate content of LFMP-treated cells increased to some extent (3.67 mmol/g protein in 48 h), while the extracellular lactate content did not change much (maintained at about 1.13 mmol/g protein), confirming that LND can indeed inhibit lactate excretion from tumor cells. The trend of lactate production in cells after FMP nanodrug treatment was similar to that of the control group, indicating that nanomaterials without AMF only have a little effect on this metabolic process. Treatment of cells with LFMP nanodrug with AMF and with TLND release capacity revealed a significant increase in intracellular lactate content over time (6.23 mmol/g protein in 48 h) and a slight increase in extracellular lactate content (1.39 mmol/g protein in 48 h). This result confirmed that LFMP + AMF can induce lactate accumulation in EMT-6 cancer cells. To describe this result more clearly, Fig. 5D summarized the ratio of intracellular to extracellular lactate content. After treatment with LFMP + AMF, the ratio of intracellular to extracellular lactate content was 5.42, significantly higher than that of free LFMP (3.08).

### In vivo ferroptosis and the related antitumor therapeutic mechanism

**Fig. 6:**
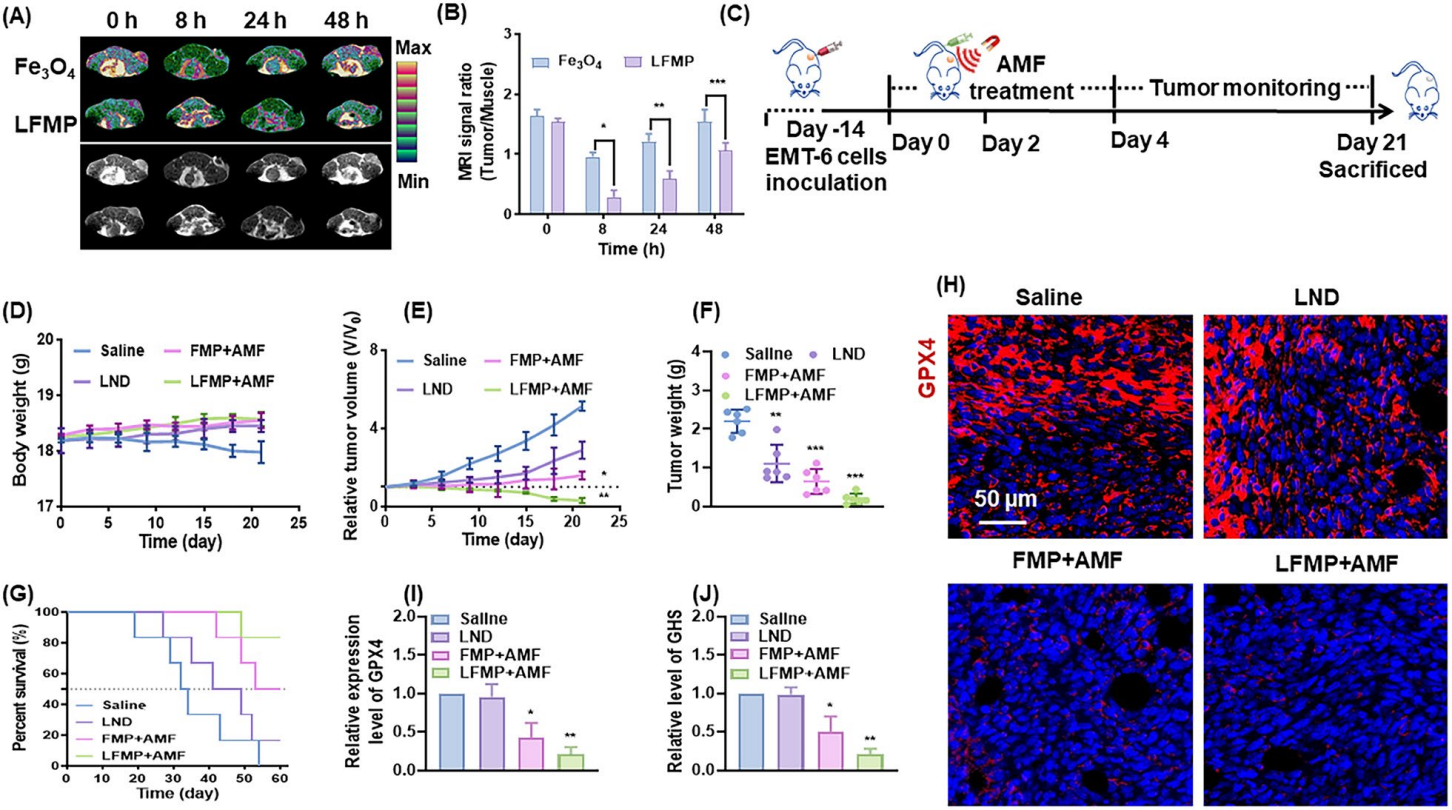
**A** *T2*-weighted MR images of the tumor (white dashed circles indicate the tumor site) at different time points after injection of Fe_3_O_4_ or LFMP. **B** Semiquantitative analysis of *T2*-weighted MR images of the tumor at different time points after injection of Fe_3_O_4_ or LFMP. **C** Schematic illustration of the in vivo therapeutic process. **D** Body weight curves of tumor-bearing mice in each group during the treatment. **E** Relative tumor volume in different therapeutics groups. **F** Average tumor weight after 28 days of various treatments. **G** Survival curves of mice in each group. **H** Immunofluorescence staining of GPX4 of tumor bearing mice treated with Saline, LND, FMP + AMF, LFMP + AMF (scale bar = 50 μm). **I** Mean fluorescence intensity of GPX4. (**J**) Relative GSH levels in tumor tissues treated with Saline, LND, FMP + AMF, LFMP + AMF. Data are shown mean ± SD, n = 5. *p < 0.05, **p < 0.01, ***p < 0.005

Considering that in vivo bioimaging was a crucial indicator for accurate tumor diagnosis and therapy, we designed the LFMP system based on MP nanoparticles containing the core of Fe_3_O_4_. LFMP, which contains MP and Fe_3_O_4_, exhibits MR imaging performance for real-time monitoring of the particle cycle process through MRI application. Firstly, we investigated the *T2*-weighted MR imaging performance in mice with cancer after LFMP treatment. As shown in Fig. 6A and B, LFMP nanoparticles accumulated in tumor locations with sustained MR imaging intensity and maintained sustainable growth from 8 to 48 h, indicating that LFMP could accumulate in the tumor region and remain for a longer duration (2 days), thereby helping to preserve the anti-cancer effect of the particles at the tumor site.

Based on the promising efficacy of LFMP on tumor cells, the tumor model on mice by injection with EMT-6 cells for 14 days were further constructed. As illustrated in Fig. 6C, the tumor-bearing mice were randomized grouping for saline, LND, FMP + AMF or LFMP + AMF administrations, and the related indexes were investigated every two days. Next, no significant influence on body weight of mice administrated with different drugs (Fig. 6D) was recorded, while the tumor volumes (Fig. 6E) and weight (Fig. 6F) were obviously reduced by LFMP + AMF treatment. Meanwhile, LFMP + AMF administration significantly improved the survival rate of mice (Fig. 6G). Additionally, the GPX4 levels and GSH activity in EMT-6 tumor tissues were also evaluated. As displayed in Fig. 6H and I, in the LFMP + AMF treatment group, an eminent decrease of the fluorescent expression of GPX4 protein in tumor sections was detected. As expected, LFMP + AMF also strikingly inhibited GSH activity in tumor tissues (Fig. 6J). These demonstrate that LFMP + AMF administration also exhibits excellent anti-tumor activity in tumor-bearing mice possibly via inducing ferroptosis with decreased GSH activity and GPX4 expression.

**Fig. 7:**
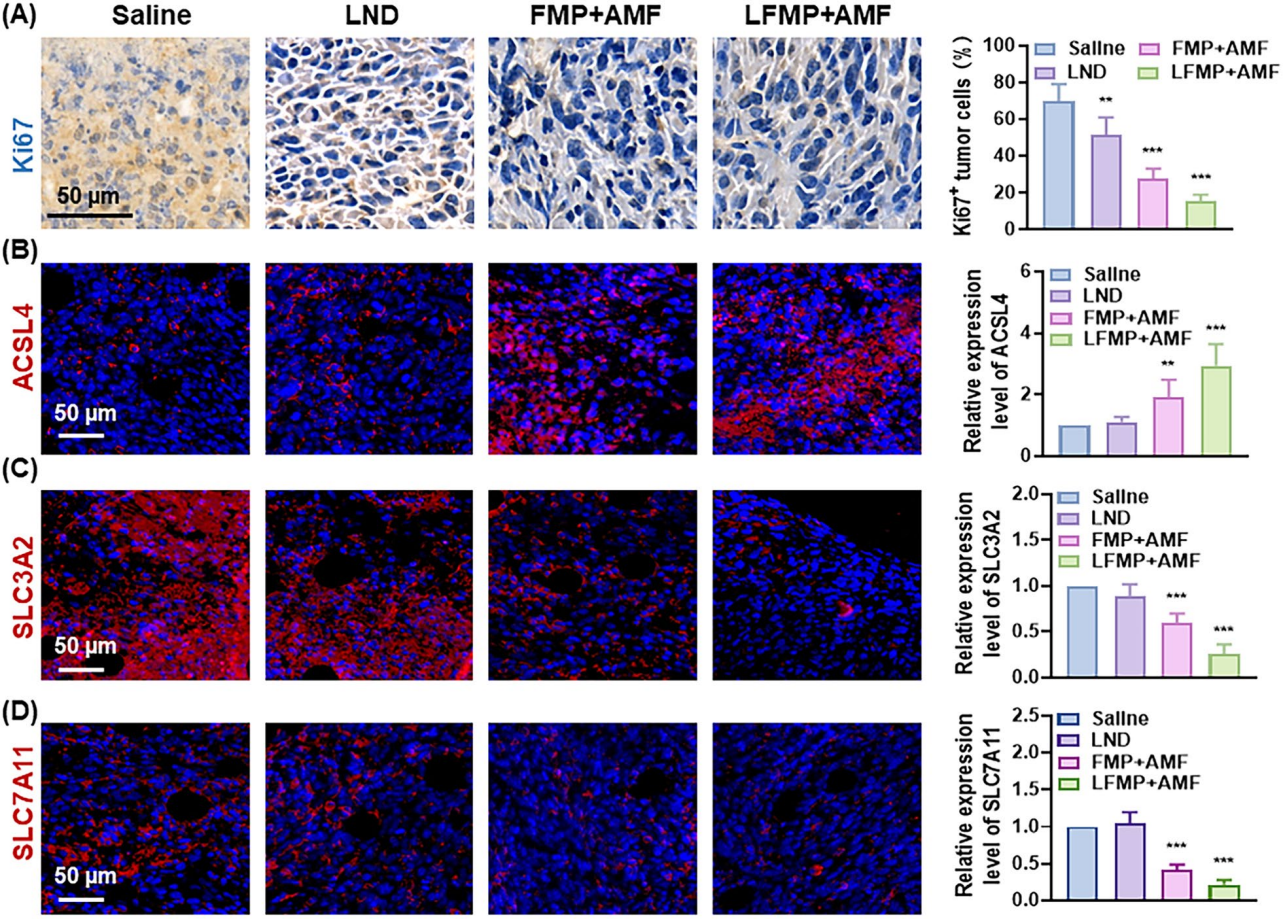
**A** Histological analysis of tumor slices by Ki 67 staining and statistics of Ki 67 positive cells after various treatments (scale bar = 50 μm). Immunofluorescence images showing the expression of ferroptosis-related proteins and relative mean fluorescence intensity, ACSL4 (**B**), SLC3A2 (**C**), SLC7A11 (**D**). (Scale bar = 50 μm). Data are shown mean ± SD, n = 5. *p < 0.05, **p < 0.01, ***p < 0.005

Meanwhile, the therapeutic mechanism of LFMP was elucidated through immunostaining of the harvested tumor tissues. The reduced expression of Ki67 in the tumor sites was assayed in the drug treatment groups, with the lowest expression observed in the LFMP + AMF group (Fig. 7A). This suggested that LFMP + AMF can effectively impede the proliferation of tumor cells. The group treated with LFMP + AMF showed evident cell apoptosis, as shown in Additional file 1: Fig. S7.

Furthermore, the connection between therapeutic effects and ferroptosis were explored. As hypothesized, an enhancement in ACSL4 expression (Fig. 7B) and a decline in SLC3A2 (Fig. 7C) and SLC7A11 (Fig. 7D) distributions in the harvested tumor section were evaluated, suggesting that LFMP + AMF exhibits an inspiring potential for antitumor effects with GSH depletion via accelerating ferroptosis. In addition, LFMP triggered no immune rejection to the host and demonstrated no liver and kidney toxicity (Additional file 1: Fig. S8). Taken together, these findings lay a solid foundation for the clinical application of LFMP in tumor treatment.

## Conclusions

In this study, we successfully constructed ferroptosis-activated, GSH-responsive nanoparticles (LFMP) based on magnetic hyperthermia to intervene in the redox homeostasis and iron homeostasis in tumor cells. LFMP nanoparticles were designed to induce a magnetothermal reaction, with the disulfide bond serving as a GSH-depleting agent and LND used to exhibit a synergistic antitumor effect with Fe_3_O_4_. This combination disrupted multi-pathway homeostasis and inhibited the proliferation of tumor cells, promoting ferroptosis. Furthermore, LFMP enhanced the anti-tumor effect of LND, potentially inducing mitochondrial apoptosis and causing ROS generation or ATP depletion in tumor cells. Importantly, LFMP allowed for MR imaging in vivo and had been demonstrated to possess favorable safety and efficacy, thereby enhancing the potential for clinical application. Overall, our work proposed the possibility of ferroptosis-based anti-tumor therapy targeting unbalanced intracellular redox homeostasis and iron homeostasis, offering feasible strategies for combination therapy in tumor.

### Supplementary Information

**Additional file 1: Figure S1**. Representative TEM images of LFMP. **Figure S2**. *T2*-weighted MR imaging (A) and transverse relaxivity (r_2_) of LFMP was examined by a clinical 3.0 T MR imaging device with a *T2* mapping sequence (B). The quantitative assay was performed by measuring the intensity of MR images using ImageJ software (C). Data shown as mean ± SD, n = 5, **p < 0.01, ***p < 0.001. **Figure S3**. Biocompatibility of different concentrations of LFMP in blood for 4 h. H_2_O as positive control, 0.9% NaCl as negative control. **Figure S4**. Representative TEM images of biodegradation behavior of LFMP in PBS solution ([GSH] = 10 mM) for 10 days (scale bar = 50 μm). **Figure S5**. Half-maximal inhibitory concentration (IC50) of LND (A), FMP (B), FMP + AMF (B), LFMP (C), and LFMP + AMF(C) in EMT-6 cells. The data are presented as the mean ± SD, n = 6. **Figure S6**. Combination index plot for drug combination of LND and FMP. **Figure S7**. TUNEL staining and the corresponding proportion of TUNEL positive cells of tumor sections after the survival experiment (Scale bar = 100 μm). The data was shown as mean ± SD, n = 6 per group, ***p < 0.001. **Figure S8**. Blood biochemical indexes and hematology parameters of the mice with different treatments. The data are presented as the mean ± SD, n = 6. **Table S1**. Physicochemical properties of FM and LFMP. **Table S2**. Hydrodynamic diameter distributions and Zeta potential of LFMP in different solutions. Data are presented as mean ± SD, n = 6.

## Data Availability

All data analyzed during this study are included in this published article (and its supplementary information files). Other raw data required to reproduce these findings are available from the corresponding author on reasonable request.
