# Supplementary material for: GSH-responsive degradable nanodrug for glucose metabolism intervention and induction of ferroptosis to enhance magnetothermal anti-tumor therapy
Source: J Nanobiotechnology. 2024 Apr 3;22:147. doi: 10.1186/s12951-024-02425-4 (PMC11321096; doi:10.1186/s12951-024-02425-4)
Supplement: Supplementary file 1 — Additional file 1: Figure S1. Representative TEM images of LFMP. Figure S2. T2-weighted MR imaging (A) and transverse relaxivity (r2) of LFMP was examined by a clinical 3.0 T MR imaging device with a T2 mapping sequence (B). The quantitative assay was performed by measuring the intensity of MR images using ImageJ software (C). Data shown as mean ± SD, n = 5, **p < 0.01, ***p < 0.001. Figure S3. Biocompatibility of different concentrations of LFMP in blood for 4 h. H2O as positive control, 0.9% NaCl as negative control. Figure S4. Representative TEM images of biodegradation behavior of LFMP in PBS solution ([GSH] = 10 mM) for 10 days (scale bar = 50 μm). Figure S5. Half-maximal inhibitory concentration (IC50) of LND (A), FMP (B), FMP + AMF (B), LFMP (C), and LFMP + AMF(C) in EMT-6 cells. The data are presented as the mean ± SD, n = 6. Figure S6. Combination index plot for drug combination of LND and FMP. Figure S7. TUNEL staining and the corresponding proportion of TUNEL positive cells of tumor sections after the survival experiment (Scale bar = 100 μm). The data was shown as mean ± SD, n = 6 per group, ***p < 0.001. Figure S8. Blood biochemical indexes and hematology parameters of the mice with different treatments. The data are presented as the mean ± SD, n = 6. Table S1. Physicochemical properties of FM and LFMP. Table S2. Hydrodynamic diameter distributions and Zeta potential of LFMP in different solutions. Data are presented as mean ± SD, n = 6. [file 12951_2024_2425_MOESM1_ESM.docx]

**Additional file**

**GSH-Responsive Degradable Nanodrug for Glucose Metabolism Intervention and Induction of ferroptosis to enhance Magnetothermal Anti-tumor Therapy**

Zhen Liao ^1 #^, E Wen ^3 #^, Yi Feng ^2 *^

^#^ These authors contributed equally to this work.

*^1^ Department of Biomedical Engineering, School of Life Science and Technology, University of Electronic Science and Technology of China, Chengdu 61173, Sichuan, P.R. China.*

*^2^* *Institute of Burn Research Southwest Hospital Third Military Medical University (Army Medical University), Chongqing,400038, China.*

*^3^ Department of Ultrasound, the Second Affiliated Hospital of Chongqing Medical University, Chongqing, China.*

**^*^ To whom correspondence should be addressed:**

Prof. Yi Feng,

Institute of Burn Research Southwest Hospital Third Military Medical University (Army Medical University)

E-mail: [fengxiaoyijoy@163.com](mailto:fengxiaoyijoy@163.com)

1. **Additional Figures**

**
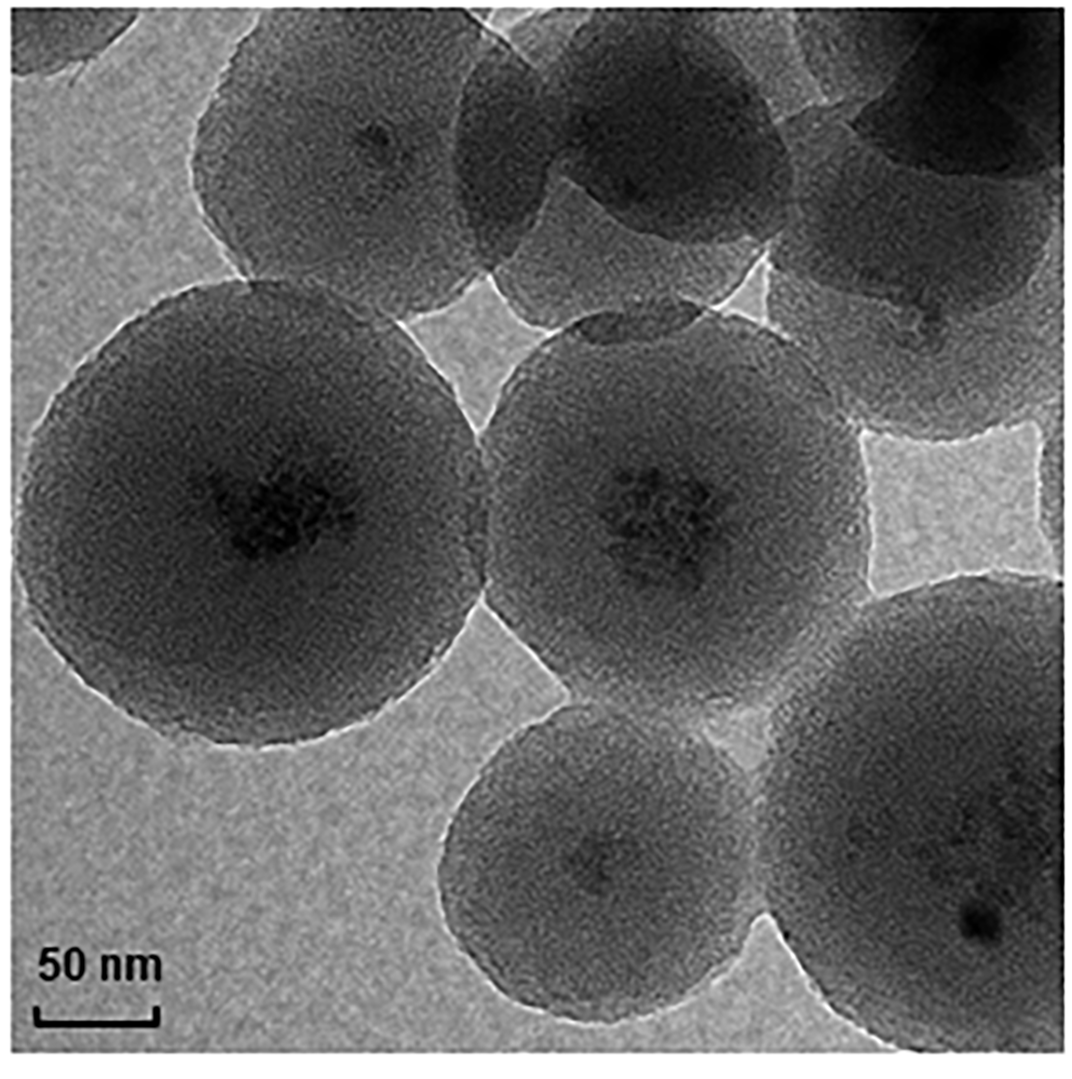
**

**Figure S1**. Representative TEM images of LFMP.


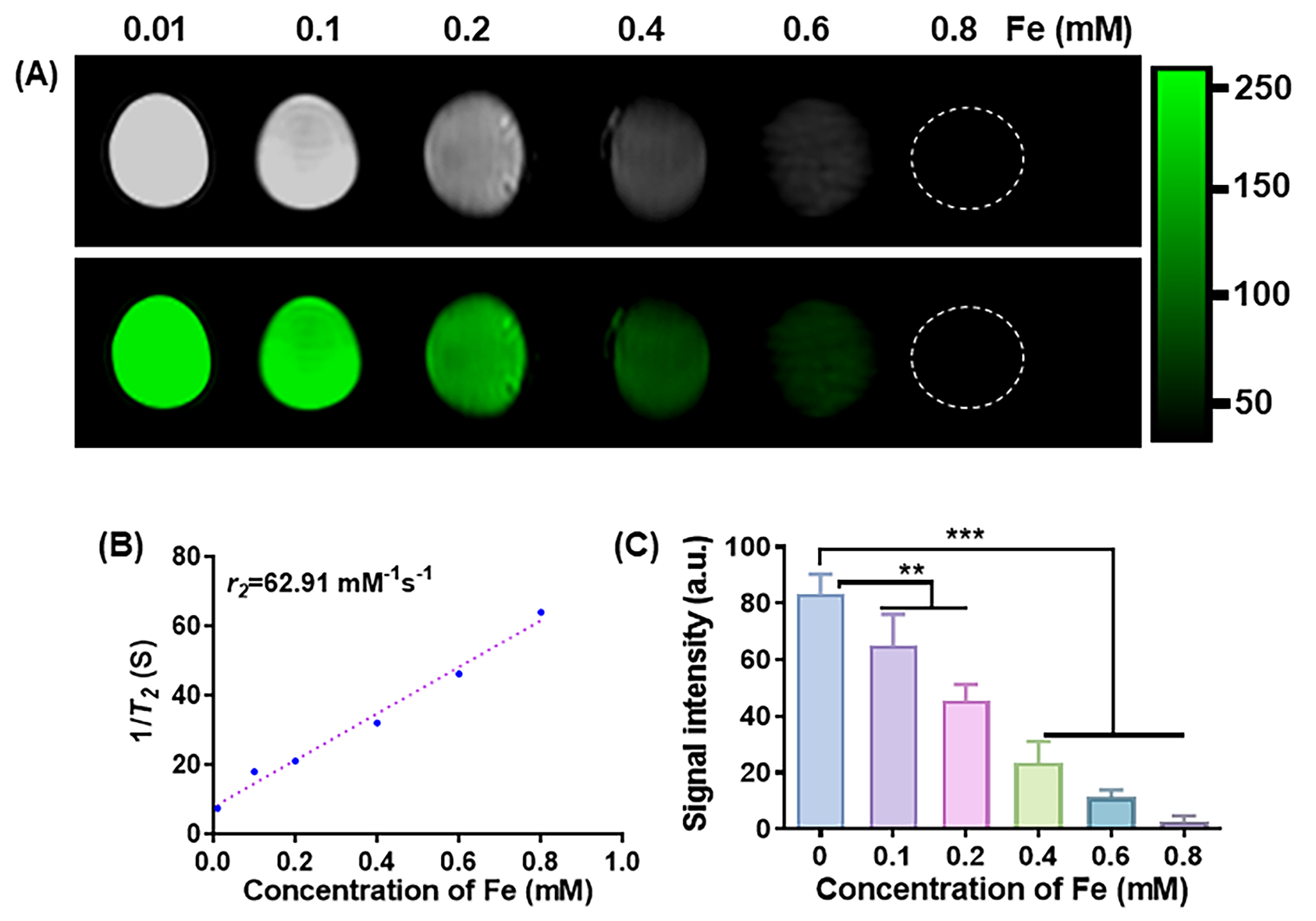


**Figure S2**. *T2*-weighted MR imaging (A) and transverse relaxivity (r_2_) of LFMP was examined by a clinical 3.0T MR imaging device with a *T2* mapping sequence (B). The quantitative assay was performed by measuring the intensity of MR images using ImageJ software (C). Data shown as mean ± SD, n=5, **p < 0.01, ***p < 0.001


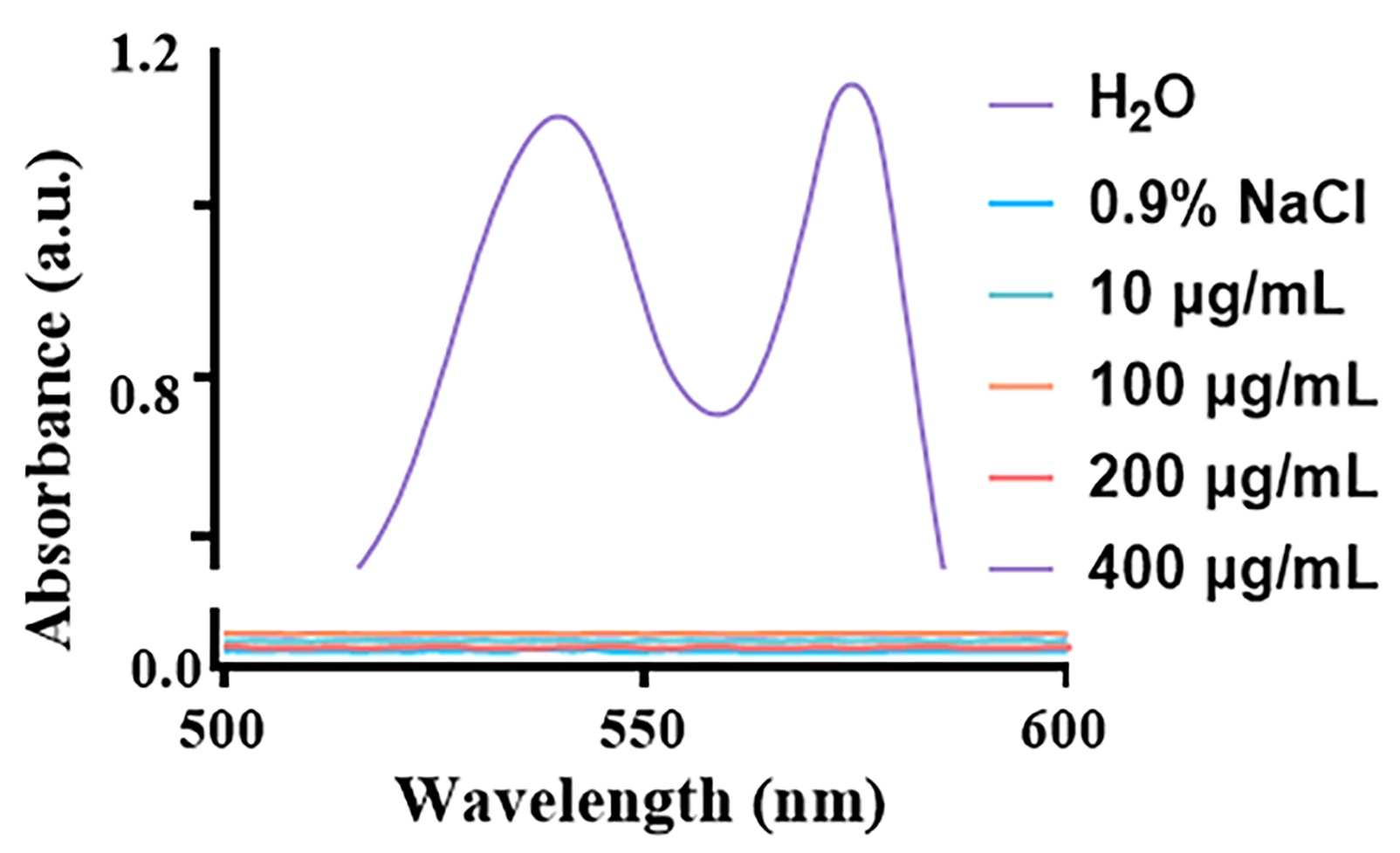


**Figure S3**. Biocompatibility of different concentrations of LFMP in blood for 4 h. H_2_O as positive control, 0.9% NaCl as negative control.


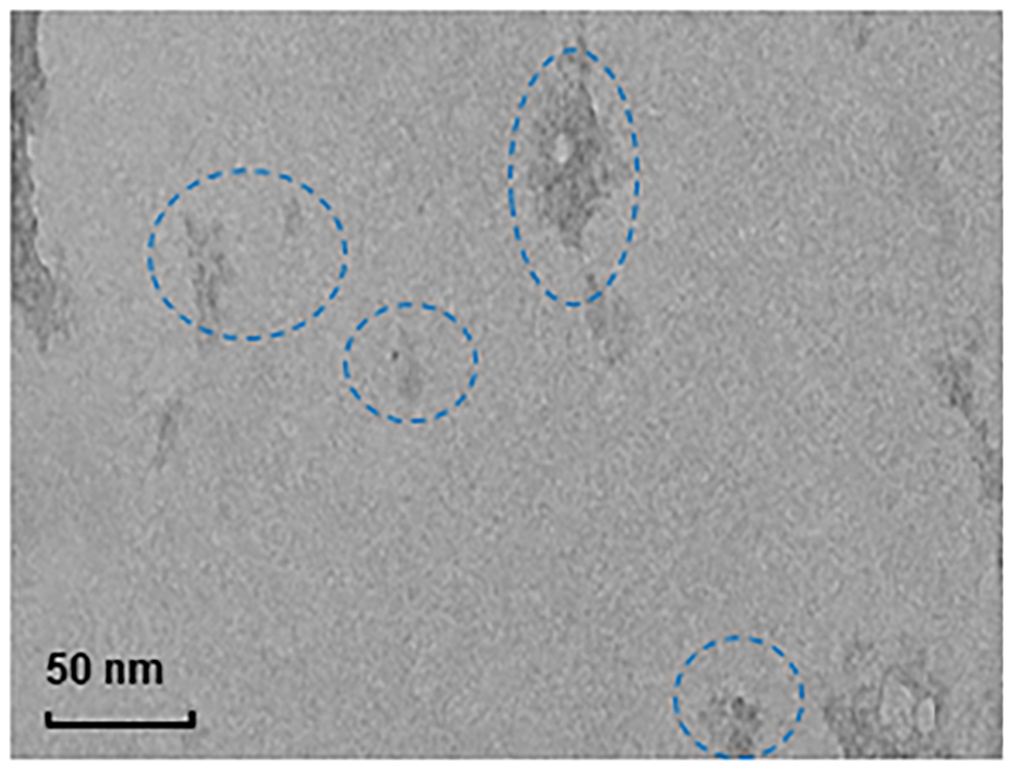


**Figure S4**. Representative TEM images of biodegradation behavior of LFMP in PBS solution ([GSH] = 10 mM) for 10 days (scale bar = 50 μm).


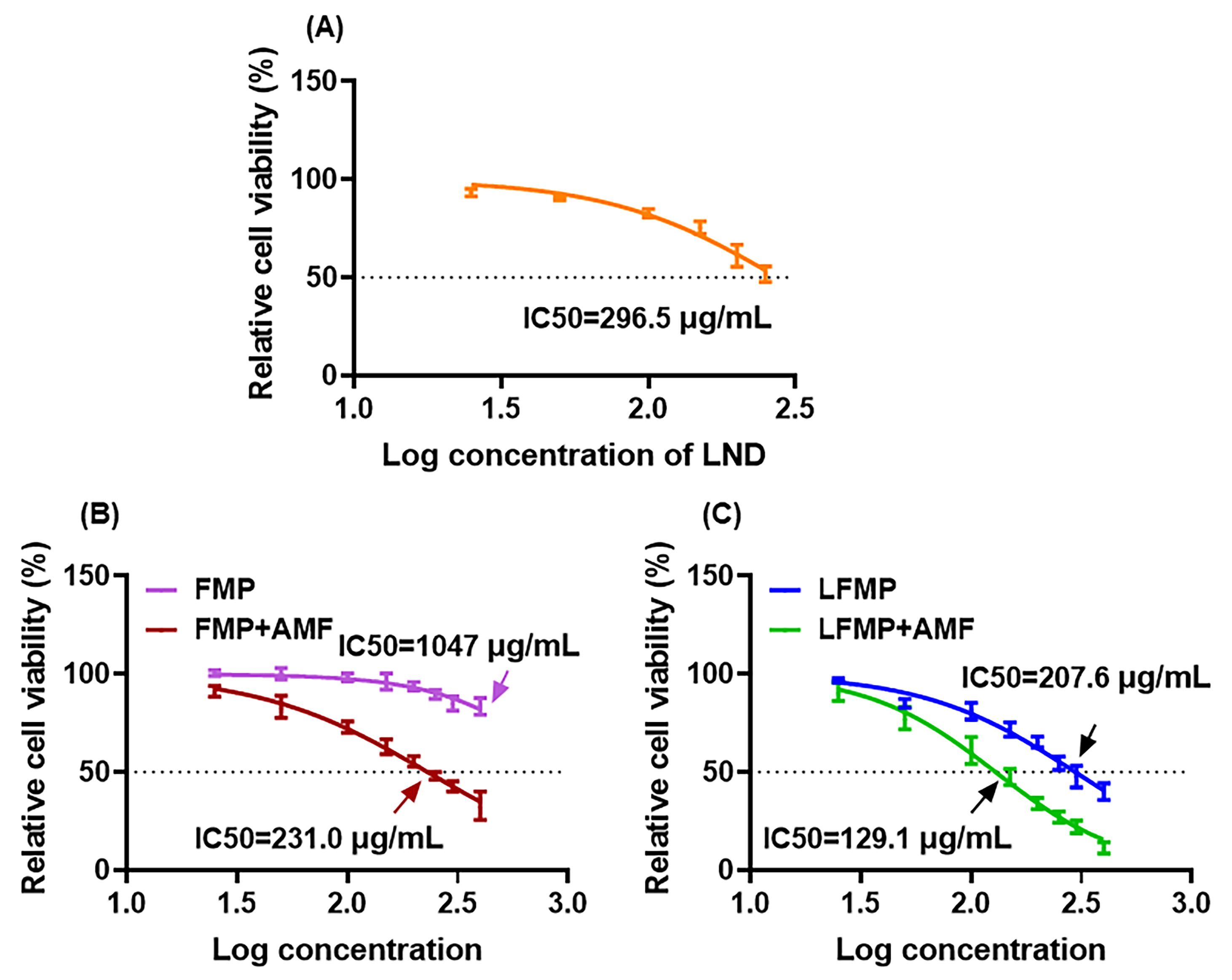


**Figure S5**. Half-maximal inhibitory concentration (IC50) of LND (A), FMP (B), FMP+AMF (B), LFMP (C), and LFMP+AMF(C) in EMT-6 cells. The data are presented as the mean ± SD, n=6.


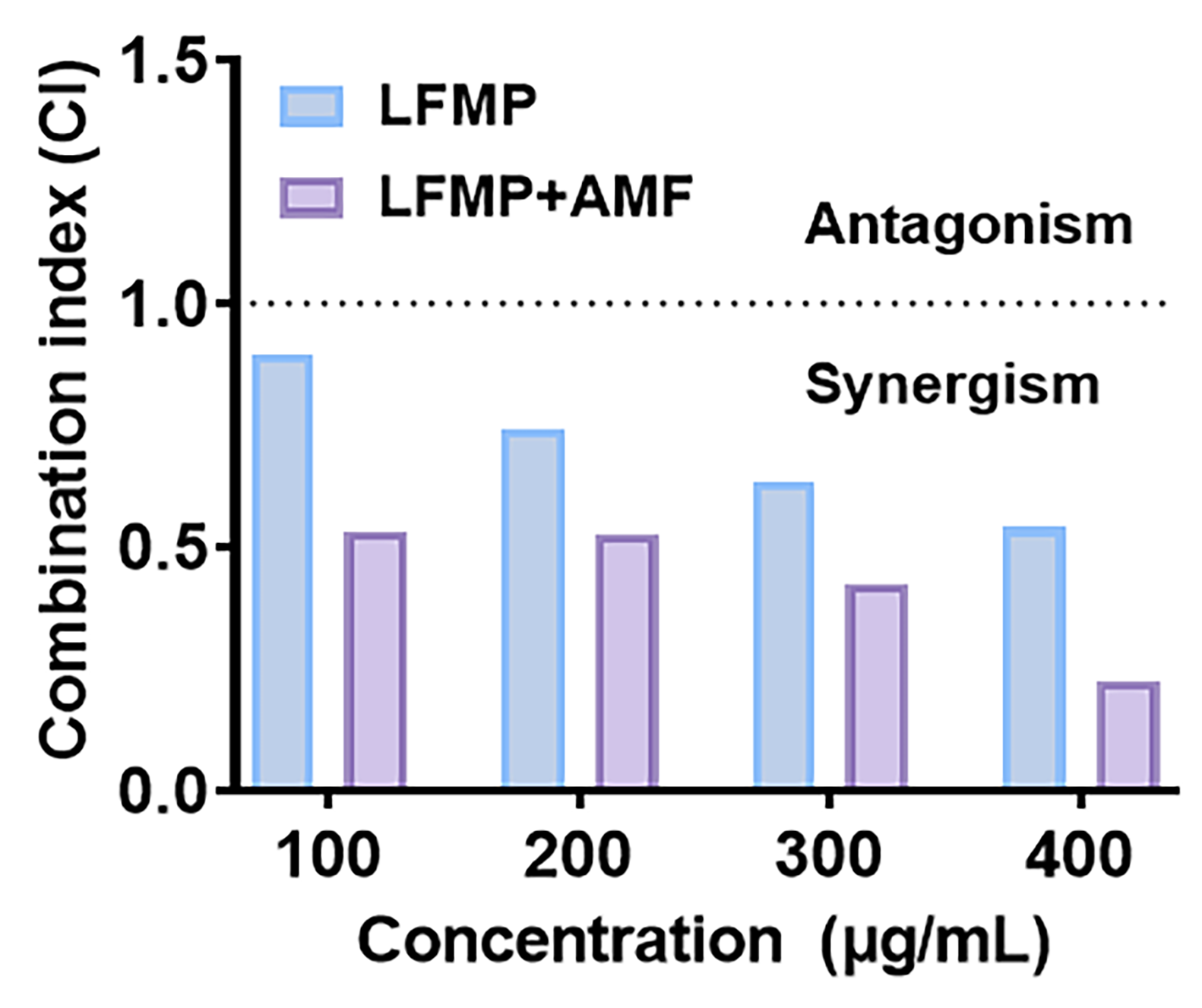


**Figure S6**. Combination index plot for drug combination of LND and FMP.


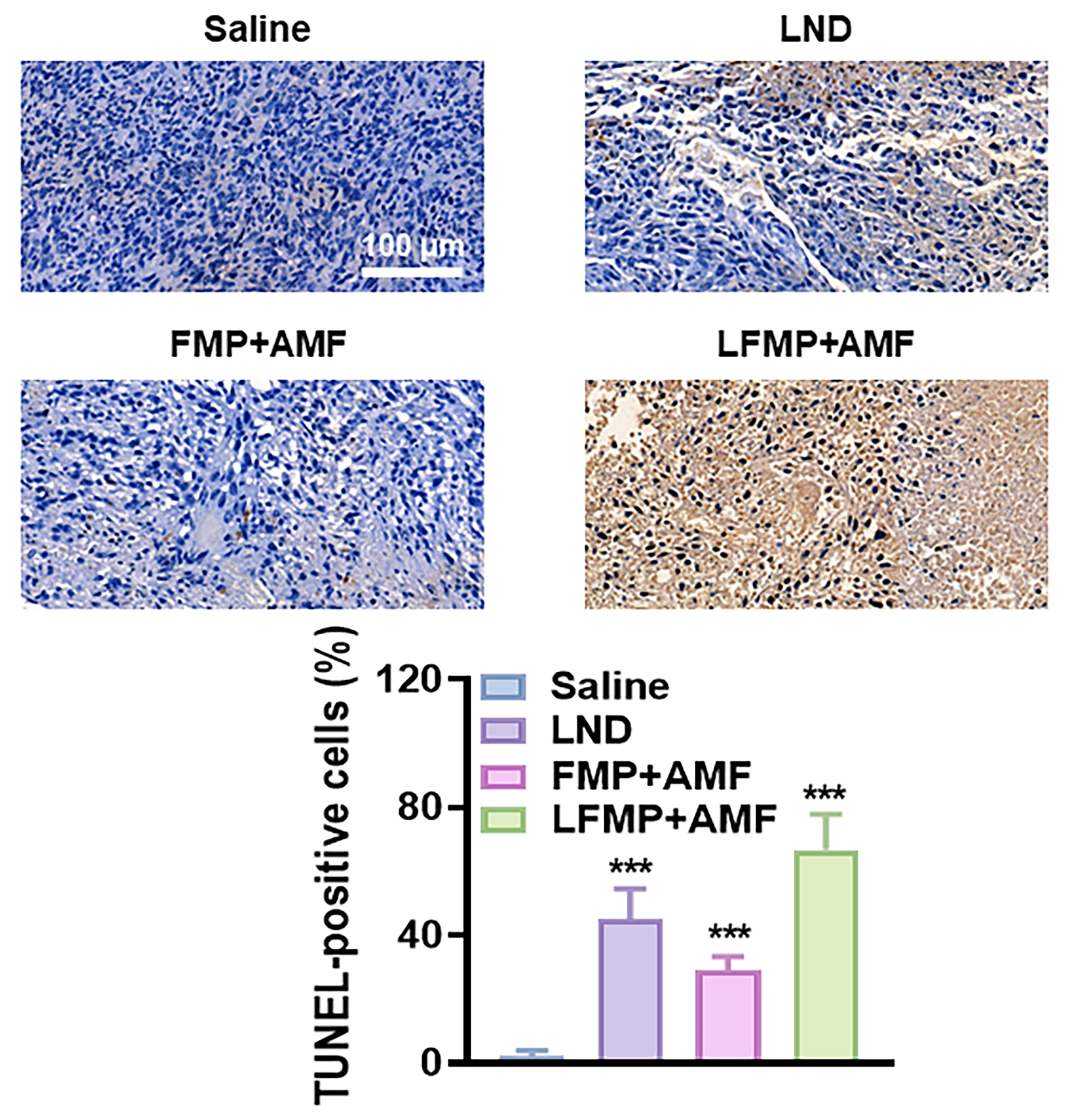


**Figure S7**. TUNEL staining and the corresponding proportion of TUNEL positive cells of tumor sections after the survival experiment (Scale bar = 100 μm). The data was shown as mean ± SD, n = 6 per group, ***p < 0.001.


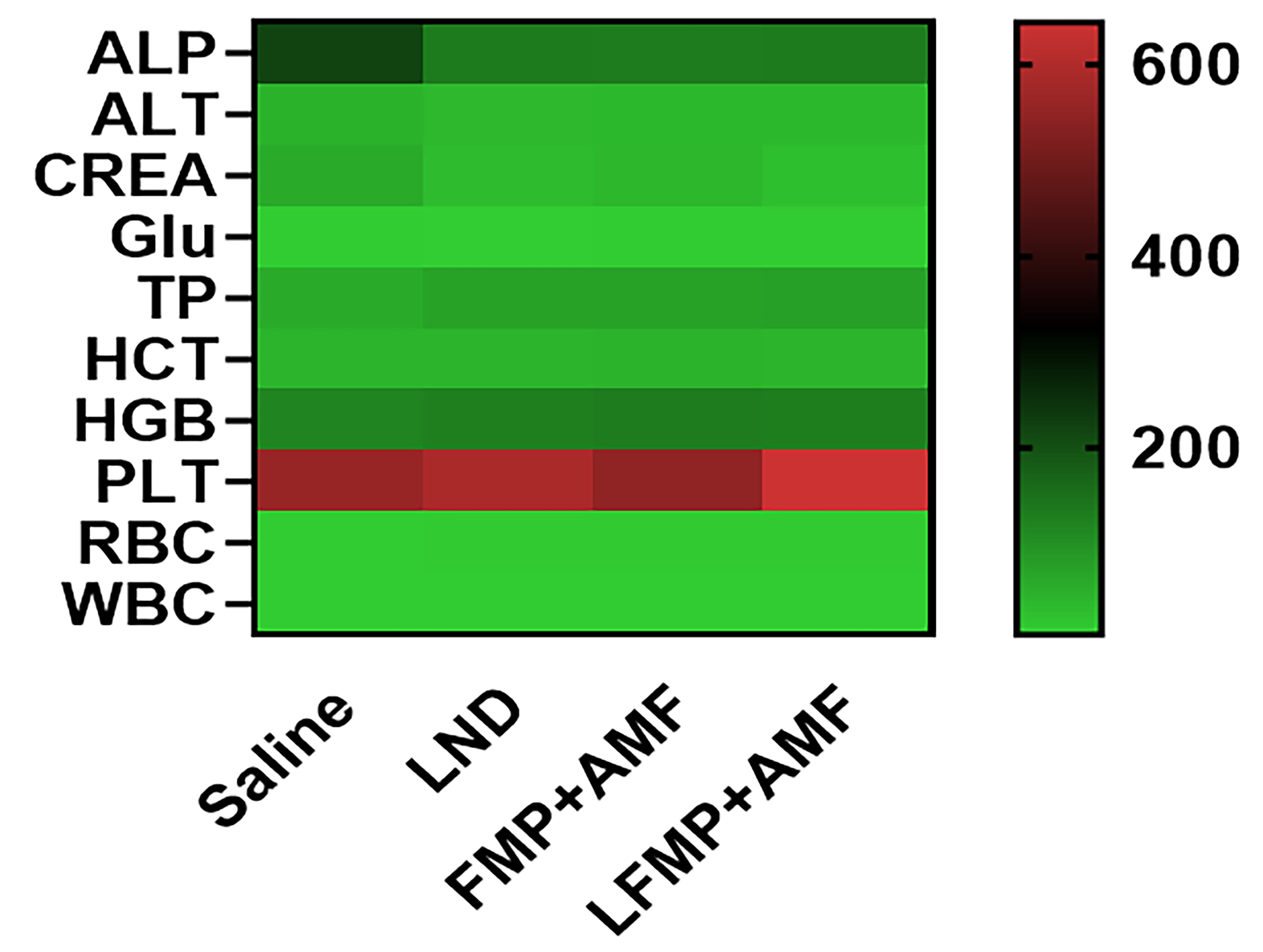


**Figure S8**. Blood biochemical indexes and hematology parameters of the mice with different treatments. The data are presented as the mean ± SD, n=6.

**2. Additional Tables**

**Table S1**. Physicochemical properties of FM and LFMP.

|  | Surface Area (m²/g) | Pore Volume (cm³/g) | Pore Size (nm) |
| --- | --- | --- | --- |
| FM | 626.47 | 0.64 | 5.38 |
| LFMP | 356.72 | 0.13 | 4.27 |

**Table S2**. Hydrodynamic diameter distributions and Zeta potential of LFMP in different solutions. Data are presented as mean ± SD, n = 6.

| Buffer | Hydrodynamic diameter (nm) | Zeta potential (mV) |
| --- | --- | --- |
| PBS | 146.54±4.31 | -5.12±0.72 |
| Saline | 152.03±4.76 | -6.77±0.28 |
| 1640 Medium | 163.22±3.95 | -7.13±1.05 |
| Fetal bovine serum | 156.64±2.03 | -6.91±0.14 |
| Complete medium | 172.11±3.02 | -4.22±0.83 |
